# Highly exposed segment of the Spf1p P5A-ATPase near transmembrane M5 detected by limited proteolysis

**DOI:** 10.1371/journal.pone.0245679

**Published:** 2021-01-28

**Authors:** Guido D. Petrovich, Gerardo R. Corradi, Carlos H. Pavan, Sofia Noli Truant, Hugo P. Adamo

**Affiliations:** 1 Facultad de Farmacia y Bioquímica, Departamento de Química Biológica, Consejo Nacional de Investigaciones Científicas y Técnicas (CONICET)-Universidad de Buenos Aires, Instituto de Química y Fisicoquímica Biológicas (IQUIFIB), Universidad de Buenos Aires, Buenos Aires, Argentina; 2 Facultad de Farmacia y Bioquímica, Cátedra de Inmunología and Instituto de Estudios de la Inmunidad Humoral Prof. Dr. Ricardo A. Margni (IDEHU), UBA-CONICET, Universidad de Buenos Aires, Buenos Aires, Argentina; University of Michigan, UNITED STATES

## Abstract

The yeast Spf1p protein is a primary transporter that belongs to group 5 of the large family of P-ATPases. Loss of Spf1p function produces ER stress with alterations of metal ion and sterol homeostasis and protein folding, glycosylation and membrane insertion. The amino acid sequence of Spf1p shows the characteristic P-ATPase domains A, N, and P and the transmembrane segments M1-M10. In addition, Spf1p exhibits unique structures at its N-terminus (N-T region), including two putative additional transmembrane domains, and a large insertion connecting the P domain with transmembrane segment M5 (D region). Here we used limited proteolysis to examine the structure of Spf1p. A short exposure of Spf1p to trypsin or proteinase K resulted in the cleavage at the N and C terminal regions of the protein and abrogated the formation of the catalytic phosphoenzyme and the ATPase activity. In contrast, limited proteolysis of Spf1p with chymotrypsin generated a large N-terminal fragment containing most of the M4-M5 cytosolic loop, and a minor fragment containing the C-terminal region. If lipids were present during chymotryptic proteolysis, phosphoenzyme formation and ATPase activity were preserved. ATP slowed Spf1p proteolysis without detectable changes of the generated fragments. The analysis of the proteolytic peptides by mass spectrometry and Edman degradation indicated that the preferential chymotryptic site was localized near the cytosolic end of M5. The susceptibility to proteolysis suggests an unexpected exposure of this region of Spf1p that may be an intrinsic feature of P5A-ATPases.

## Introduction

P5-ATPases are primary active transporters present in all eukaryotic cells [1–3]. Dysfunction of P5-ATPases affects the function of ER, lysosomes, peroxisomes, and mitochondria, in parallel with changes in the lipid and metal ion homeostasis [4–15]. According to the conservation of the amino acid sequence the P5-ATPases are classified into subgroups A and B [16, 17]. Humans have one gene coding for a P5A ATPase, the ATP13A1 protein and four genes coding P5B ATPases ATP13A2-A5. Lower eukaryotes like the yeast *Saccharomyces cerevisiae* have one P5A-ATPase, Spf1p and only one P5B-ATPase, Ypk9p. A number of neurological disorders like the juvenile onset Parkinson disease-9 or Kufor-Rakeb syndrome, neuronal ceroid lipofuscinosis and spastic paraplegia have been associated with mutations in the lysosomal P5B-ATPase ATP13a2 [18–20]. Recently ATP13A2 has been shown to be a polyamine transporter [21]. The P5A-ATPases have been proposed to transport metal ions like Ca^2+^, Mg^2+^ and Mn^2+^ and sterols [6, 9, 11, 14]. However, recent data shows that P5A-ATPases function as transmembrane helix dislocases suggesting that the transported substrate is of peptidyl nature [22, 23]. The Spf1p, the best characterized P5A-ATPase, is a protein of Mr~135 kDa localized in the yeast ER. Purified preparations of recombinant Spf1p have been shown to be capable of ATP hydrolysis and to form maximal levels of the characteristic P-ATPase catalytic phosphoenzyme in the absence of metal ions, with the exception of Mg^2+^ [6, 14, 24, 25].

The comparison of primary sequences allows the identification of the characteristic P-ATPase actuator (A), phosphorylation (P) and nucleotide binding (N) domains, plus the canonical 10 transmembrane segments (M1-M10) [16]. The overall identity of amino acid sequence between the P5 group and other P-type ATPases is relatively low, in particular in the transmembrane segments, a fact probably related to their transported substrate specificity. In addition the P5-ATPases have a unique N-terminal region, which in the P5A subgroup includes two extra transmembrane segments called “Ma” and “Mb” (region N-T, Spf1p amino acids 1–72). Also a unique feature of the P5A-ATPases is the presence of an extended segment connecting the C-terminus of P domain with the cytosolic portion of M5 (region D, Spf1p amino acids 840–956). Here we call this segment “D region” because is predicted to have a disordered structure towards its C-terminus [26]. Structural information about Spf1p that became available during the revision of this work shows that the first ten amino acids of this segment is an helix of elongated shape that was called “arm-like” domain and that extends from the core of the protein toward the surface of the membrane [22].

Limited proteolysis is a low resolution and yet a powerful approach to the structure of proteins. The location of the primary sites of hydrolysis of the peptide chain by proteases gives information about surface exposed segments, domain boundaries and substrate sites [27]. In general, the actual cleavage sites of a native protein are determined more by the tertiary structure than by the specificity of the protease [28]. Limited proteolysis of P-ATPases has been a particularly useful technique for the study of the topology, domain function and conformational changes of P-ATPases [29–38], and has revealed the presence of non-essential regulatory domains in several members of the family, i.e. the auto-inhibitory domains of the plasma membrane Ca^2+^ pumps (PMCA), the yeast plasma membrane H^+^-ATPase (Pma1p) and the lipid flippase Drs2p [39–42].

Here we used limited proteolysis to gain information on the structure of Spf1p. We found that a short exposure of Spf1p to trypsin and proteinase K cleaved portions of the N and C-terminal ends of the molecule and inactivated the enzyme. Unexpectedly, digestion with chymotrypsin produced an initial cut of the Spf1p protein in the region of the large cytosolic loop near transmembrane segment M5. In the presence of lipids, the fragments generated by limited chymotrypsin digestion of Spf1p were still able to hydrolyze ATP and form the catalytic phosphoenzyme.

## Materials and methods

### Chemicals

Polyoxyethylene-10-laurylether (C_12_E_10_), Polyethylene Glycol- p-isooctylphenyl Ether (Triton X-100), L-α- phosphatidylcholine from soybean (Sigma P5638), ATP (disodium salt, vanadium-free), SDS, yeast synthetic drop-out medium supplement without leucine, yeast nitrogen base without amino acids, dextrose, enzymes, α-Chymotrypsin from bovine pancreas (Sigma C3142), Trypsin, Proteinase K, V8 protease and all other chemicals were obtained from Sigma. Tryptone and yeast extract were from Difco. [γ^32^P]-ATP was provided by PerkinElmer Life Sciences (Boston, MA). Salts and reagents were of analytical reagent grade.

### Yeast strains, transformation, and growth media

Recombinant Spf1p was expressed in *S*. *cerevisiae* strain DBY 2062 (MATα his4-619 leu2-3,112) as described previously [24]. Yeast cells were transformed with the pMP625 vector containing a Leu marker and the promoter of plasma membrane H^+^-ATPase from *S*. *cerevisiae*. Yeasts were transformed using the lithium acetate/polyethylene glycol method with plasmids coding for Spf1p and GFP-Spf1p described in [25]. The cells were grown in complete medium (0.75% yeast extract, 1.13% tryptone, 2.2% dextrose), and transformants were selected for their ability to grow in the absence of leucine on plates containing 6.7% yeast nitrogen base without amino acids (YNB), 0.67% complete supplemented medium minus Leu (Leu-), 2.2% dextrose, and 1.5% agar. Spf1p-GFP was cloned by homologous recombination into a 2 μ GFP-fusion vector following the protocol described by Drew et al [43].

### Membrane isolation and purification of GFP-Spf1p, Spf1p and Spf1p-GFP

Total membranes from eight liters of yeasts expressing the recombinant protein were obtained as previously described [24, 44]. Briefly, the microsomal membranes were solubilized at 4°C for 30 min by adding 2 g of Triton X-100/g of total membrane proteins and the supernatant was loaded onto a column with 2-ml nickel-nitrilotriacetic acid—agarose resin (Qiagen). The purification was carried out following the protocol for the production of “pure Spf1p” previously described [44]. Finally, the protein was eluted in purification buffer containing 0.005% C_12_E_10_ and 150 mM imidazole. The eluate fractions of higher protein content were pooled, aliquoted, and kept in liquid N2.

### Limited proteolysis assay

Enzymatic digestion was performed in the following standard reaction mixture, 20 mM Tris-HCl (pH 7.4 at 28°C), 0.5 mM EGTA, MgCl_2_ to give 2 mM free Mg^2+^ and 5 mM NaN_3_. The amount of protein indicated in each experiment was reconstituted with PC at a ratio protein: lipid of 1:30 (w/w). The suspension was thoroughly mixed and preincubated for at least 5 minutes on ice before being added to the reaction mixture. All solutions were equilibrated 10 minutes at 28°C, after, proteolysis started by the exposed to Chymotrypsin (Chy), Trypsin (Try), Proteinase K (Prt K) or Endoproteinase Glu-C (V8). All proteases were resuspended in MQ water. The final reaction volume was 250 μl and the volume of protease suspension added did not exceed 2 μl. Proteolytic reaction was stopped by addition of 10% TCA at a total volume. When the limited proteolysis assay was followed by the measurement of the fluorescent GFP-associated peptides, the total reaction volume was 30 μl and the reaction was stopped using either 1:40 aprotinin or 0.5 mM phenylmethylsulfonyl fluoride (PMSF).

### ATPase activity

The ATPase activity was estimated at 28°C by measuring the release of Pi from ATP using the colorimetric method of Baginski [45] as previously described [44]. Reaction medium was identical to that used for proteolysis and contained 1 μg of Spf1p supplemented with PC plus 3 mM ATP. ATP solution was prepared from the disodium salt and neutralized at pH 7.4.

### Phosphorylation of Spf1p and Spf1p-GFP

The phosphorylation reaction was performed as previously described [24]. Briefly, 3 μg of purified protein was phosphorylated at 4°C in 0.25 ml of standard reaction mixture. The reaction was started by the addition of 0.5 μM [γ^32^P]-ATP and stopped after 30 s with 10% ice-cold TCA. After 20 min on ice the samples were centrifuged for 10 min at 10,000 × g. The pellet made of denatured proteins was washed with 1 ml of cold water and was centrifuged again. The precipitated proteins were suspended in sample buffer and separated by acidic SDS-PAGE. The gels were dried, and the radioactivity was detected using a Storm Molecular Image System. For dephosphorylation, the reaction was initiated by the addition of 0.5 mM of cold ATP-Mg and continued for 5 or 30 s on ice before the addition of 10% ice-cold TCA.

### Protein assay, SDS-PAGE, in-gel fluorescence and Western blotting

The protein concentration was initially estimated by the method of Bradford [46] and then corrected according to the intensity of the bands after SDS-PAGE on an 9% acrylamide gel using bovine serum albumin standard. The peptides generated by limited proteolysis were separated by SDS-PAGE according to Laemmli [47] and were either stained with Colloidal Coomassie blue or subjected to Western blot. Nonspecific binding was blocked by incubating the PVDF membranes overnight in a solution of 1% fat-free dry milk. The membranes were incubated for 1 h with polyclonal anti Spf1p, washed three times in PBS with 0.05% Tween 20 and stained with a biotinylated anti-mouse IgG antibody and avidin-peroxidase conjugate. The molecular weight of generated fragments was determined comparing their position with that of a molecular weight marker (M). In-gel fluorescence was detected by ImageQuant LAS 500 after rinsing the gel with water. Polyclonal sera anti-Spf1 was obtained by intramuscular immunization of Balb/c mice with 5 μg of protein Spf1 mixed with incomplete Freund’s adjuvant. Boosts were administered on days 7, 14 and 28. The anti-sera obtained on day 35 were diluted and tested by ELISA and Western Blot. All the animals were manipulated following the ethics standards of the Universidad de Buenos Aires, Facultad de Farmacia y Bioquímica.

### Mass spectrometry

The proteins were separated by SDS-PAGE and stained with colloidal coomassie blue. The colored bands were cut, washed out, and treated with 10 mM DTT for 30 min at room temperature and with 80 mM iodoacetamide in darkness at room temperature. The proteins were digested “in gel” with 25 ng/μL trypsin (Promega, Madison, WI) in digestion buffer (50 mM NH4HCO3) at 37°C overnight. The sample was loaded in a μZipTip C18 and eluted with a minimal volume of matrix solution—10% mg/mL (α-Cyano-4-hydroxycinnamic acid in acetonitrile:methanol:TFA 40:30:0,1 v/v)—onto the MALDI target plate. The extracted tryptic peptides were subjected to MS and MS/MS analysis in an MALDI TOF TOF ABI4800 plus mass spectrometer. Peptides were identified by searching against the SwissProt database using the MASCOT search engine (http://www.matrixscience.com).

### Edman sequencing

The stained, electroblotted samples were washed successively with methanol, water and methanol, cut and introduced in the reactor chamber of an automatic protein sequencer–PPSQ31A model from Shimadzu–to perform Edman degradation. Specific blot cycles were used for couple, cleavage and extraction of the anilinothiazolinones. The corresponding PTH-AA was analyzed by RP-HPLC-UV.

### Data analysis

Representative experiments are shown. Limited proteolysis was performed three to five times with independent purified protein preparations. The quantitation of the intensity of the bands and plotting was done using Image J and SigmaPlot 10 scientific data analysis and graphing software (Systat Software Inc., CA) for Windows.

## Results

### SDS-PAGE of Spf1p peptides produced by proteolysis

**Fig 1:**
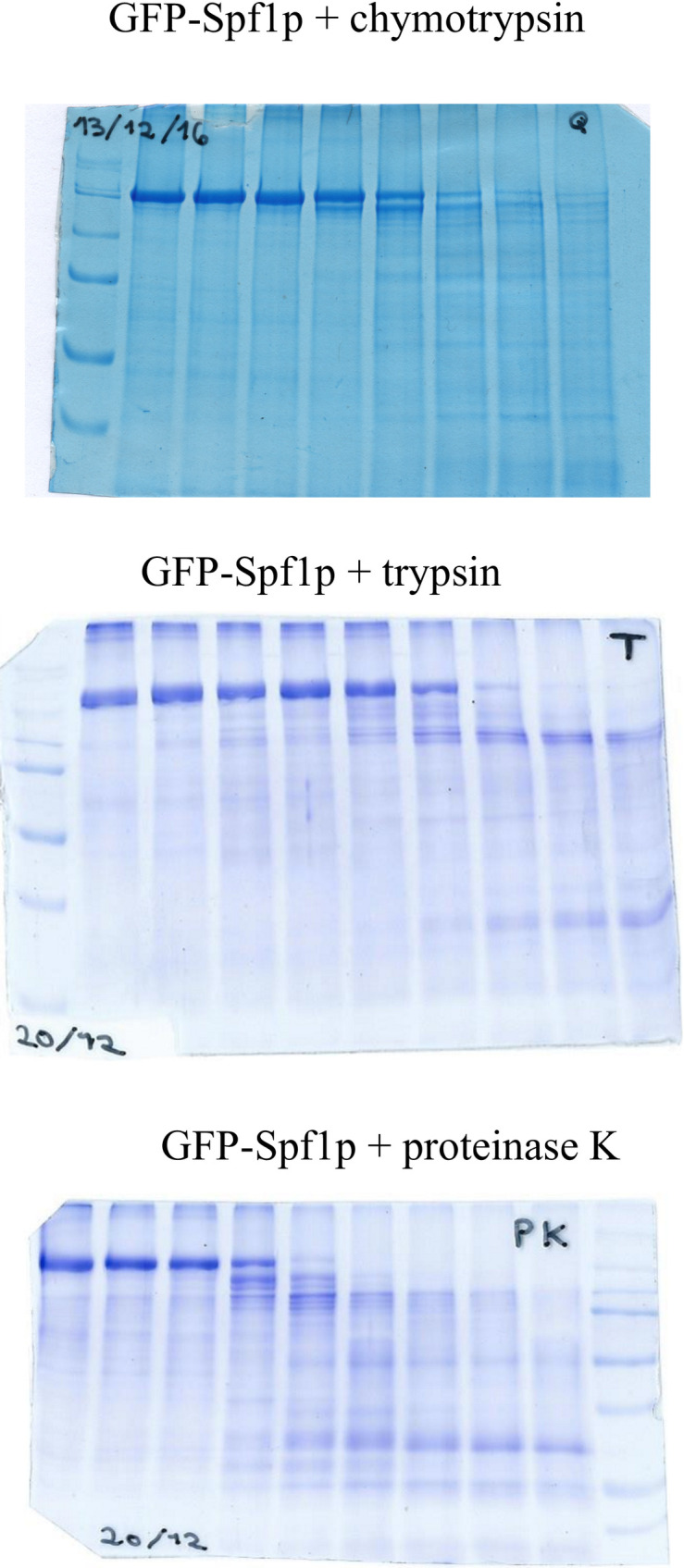
Limited proteolysis of GFP-Spf1p. 2.5 μg of GFP-Spf1p was treated with the indicated protease as described under “Materials and methods”. After the time indicated on top of each lane the proteolysis was stopped by the addition of TCA and the samples submitted to SDS-PAGE on a 9% gel and stained with Colloidal Coomassie Blue. GFP-Spf1p: protease ratio (*w/w*) was 20:1 *(A)* chymotrypsin, *(B)*, trypsin, *(C)*, proteinase K. The “0” min were processed adding TCA before the protease. The arrows indicate the estimated Mr according to the migration.

The fusion of GFP to a protein is useful as a tool for limited proteolysis analysis because GFP is relatively resistant to proteases and it may generate easily identifiable fluorescent peptides derived from the target protein. Importantly, the fusion of GFP at the N or C terminal ends of Spf1p does not impair the ATPase activity nor the formation of the catalytic phosphoenzyme suggesting that the structure of Spf1p is not grossly affected [2, 14, 24, 25]. We exposed purified Spf1p containing GFP at the N-terminus (GFP-Spf1p) to different proteases, separated the products by SDS-PAGE and visualized the peptides by Coomassie blue staining and fluorescence. A short digestion of GFP-Spf1p (Mr~ 164 kDa) with chymotrypsin generated a main product of an estimated size of 140 kDa (Fig 1). We called the cleavage site generating this fragment “Chy1”. Further digestion produced peptides of 125, 82, 54 and 40 kDa and after 60 min of chymotrypsin digestion very little of the GFP-Spf1p remained intact and numerous bands were detected. When GFP-Spf1p was digested with trypsin several bands were observed with a predominance of bands of 100 kDa and 35 kDa. The digestion of GFP-Spf1p with proteinase K also produced fragments of about 140–100 kDa that disappeared at long digestion times in parallel with the increase of the intensity of bands in the region of 50–25 kDa. These results show that despite the specificity of the particular protease, the initial proteolytic digestion of GFP-Spf1p involved the cleavage of the protein ends generating large fragments, that judging by its size, would include the catalytic core.

**Fig 2:**
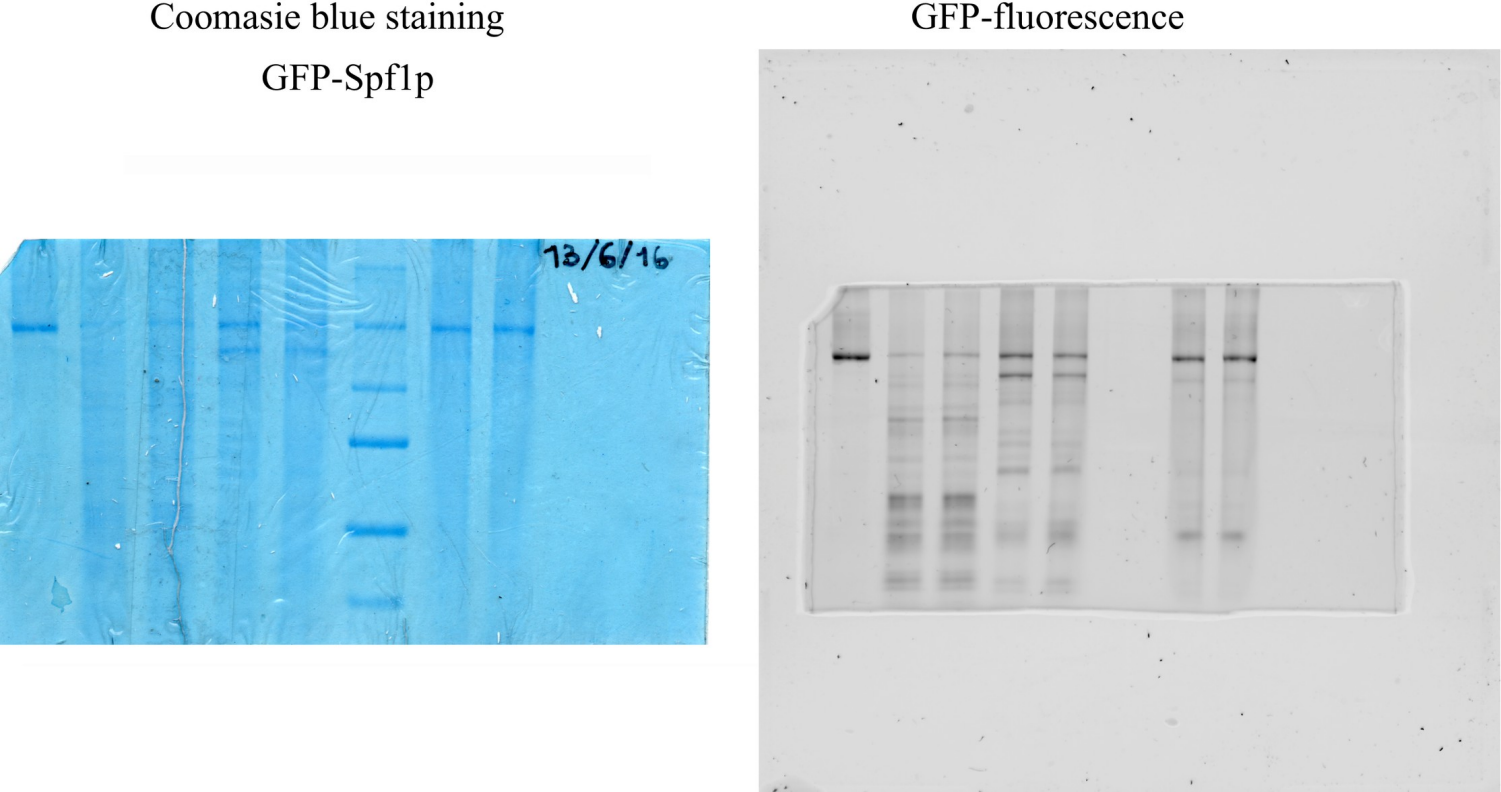
In-gel fluorescence analysis of the GFP-Spf1 digests. 1.5 μg GFP-Spf1 was exposed for 5 min at 28°C to the action of the indicated protease as described under “Materials and methods”. GFP-Spf1p: protease ratio *(w/w)* was *Try*, trypsin: (5:1), *Chy*, chymotrypsin (20:1) and *Prt K*, proteinase K (20:1), and the reaction was stopped by the addition of 12 μg or 3 μg of aprotinin for trypsin and chymotrypsin respectively. Proteinase K digestion was stopped by the addition of 0.5 mM PMSF. The panel on the left shows the Coomasie blue stained gel and on the right the measurement of GFP fluorescence. Two lanes from duplicate proteolysis reactions for each protease are shown.

**Fig 3:**
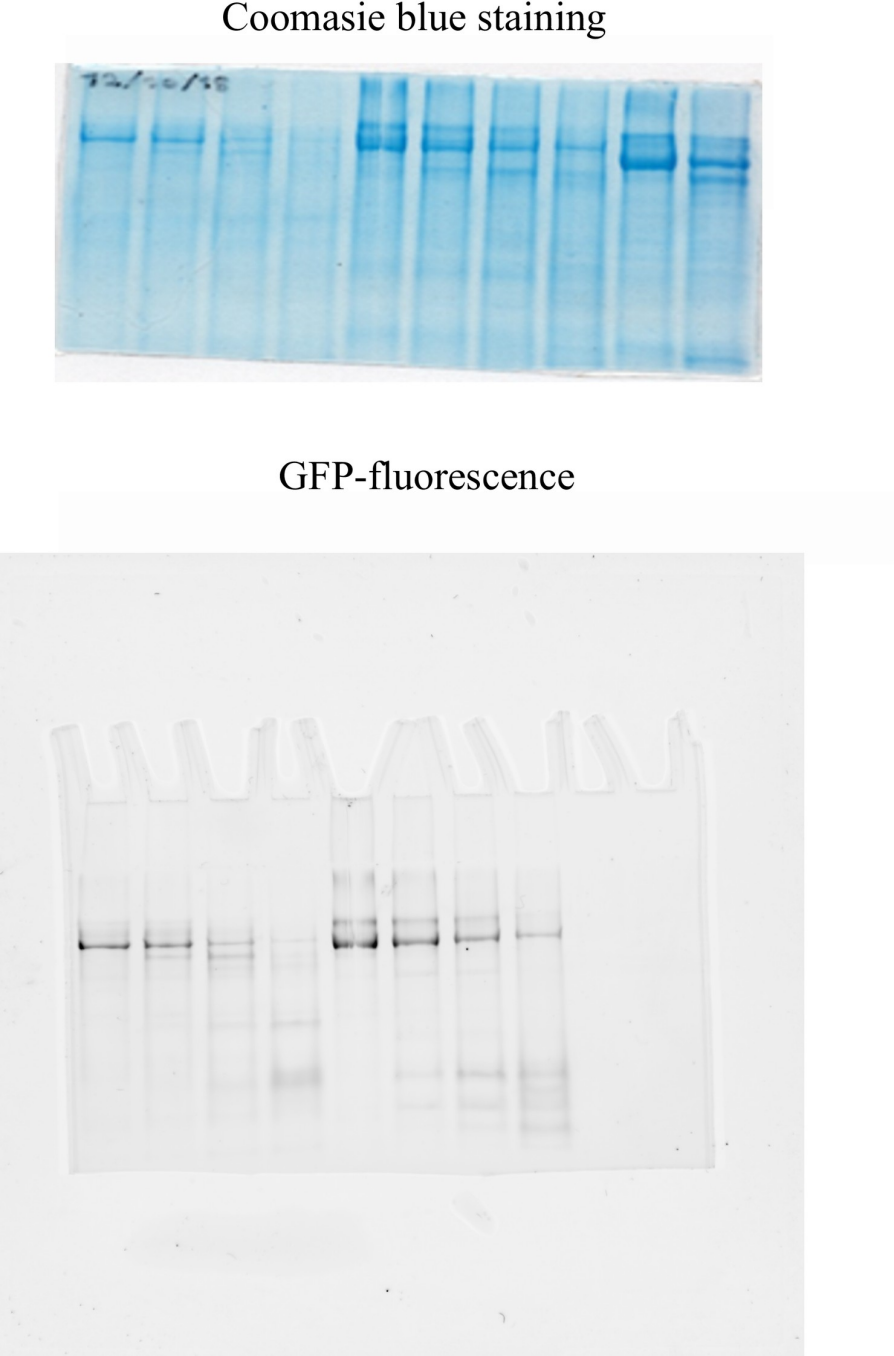
Comparison of the chymotryptic digestion pattern of GFP-Spf1, Spf1p-GFP and Spf1p. Spf1p and the Spf1p GFP fusion protein indicated in the figure were digested with chymotrypsin at a ratio protein: protease (w/w) of 20:1. After the time indicated on top of each lane, the proteolysis was stopped by adding 5 μg of aprotinin. The samples were electrophoresed in a 9% SDS-PAGE and revealed by Colloidal Coomassie staining (*left panel*) or GFP fluorescence (*right panel*). The arrows indicate the migrations of the GFP-Spf1p fragments produced by the cut at Chy1 and the equivalent non-fluorescent fragment generated from Spf1p-GFP and Spf1p.

Because in GFP-Spf1p the GFP is located at the N-terminus of Spf1p, the fate of this portion of the protein during proteolysis can be determined by following the GFP fluorescence. For that purpose, and in order to preserve the GFP fluorescence, the proteolytic digestion was stopped by protease inhibitors instead of TCA precipitation. In these conditions the GFP structure is partially conserved and the migration of GFP-containing peptides may not accurately reflect their actual relative molecular mass (Mr). Despite this difficulty, we observed that the digestion of GFP-Spf1p with trypsin rapidly generated fast migrating fluorescent fragments, suggesting that short N-terminal peptides containing the GFP were produced (Fig 2). These peptides were likely to include GFP (27 kDa) and a portion of the N-terminal region of Spf1p (N-T region). In contrast, the 140 kDa peptide generated by the chymotrypsin cleavage of GFP-Spf1p (Chy1 site) exhibited intense fluorescence, and only at longer digestion times smaller fluorescent fragments were detected (see also S1 Fig). Like trypsin, proteinase K rapidly produced short fluorescent peptides migrating at the bottom of the gel. These results suggest that, in contrast with trypsin and proteinase K, chymotrypsin cleaved the GFP-Spf1p preferentially at a site located towards the C-terminal region of the protein. In order to confirm this idea, we digested with chymotrypsin a version of Spf1p containing the GFP at the C-terminus (Spf1p-GFP). As shown in Fig 3, the main product of the Spf1p-GFP cleavage of was a non-fluorescent peptide, in agreement with the idea that the cleavage at the Chy1 site lead to the loss of the C-terminal region containing the GFP. Importantly, the chymotrypsin digestion of the non-GFP fused Spf1p also originated a peptide similar to that produced by Spf1p-GFP indicating that the chymotryptic susceptibility of the cut at Chy1 was an intrinsic feature of Spf1p and not produced by the GFP fusions.

### Proteolysis of Spf1p in yeast microsomal membranes

**Fig 4:**
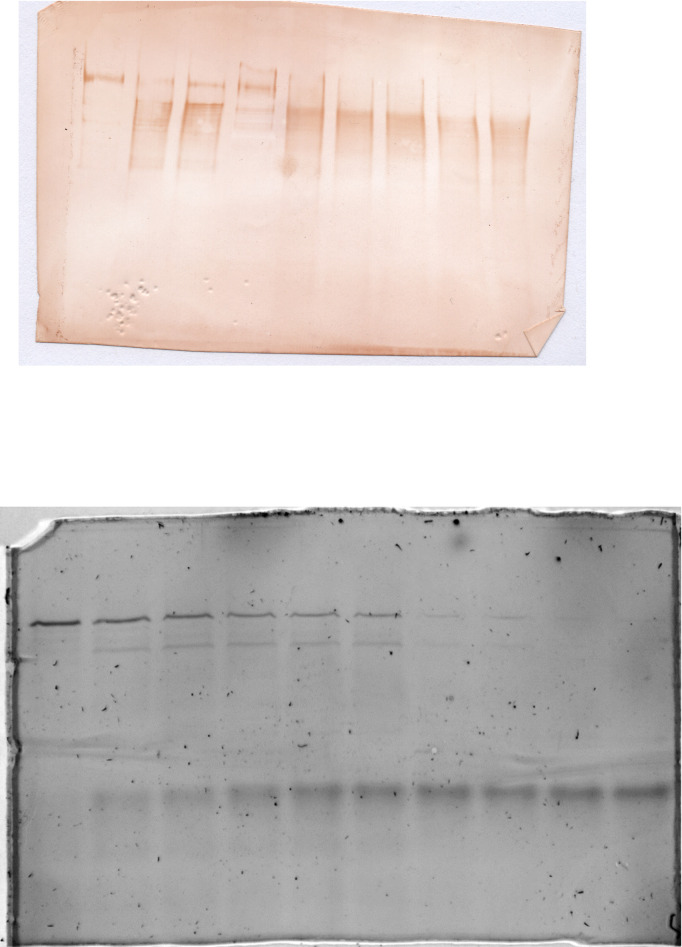
Chymotryptic digestion of detergent-purified Spf1p and Spf1p from yeast membranes. *Top panel*, 0.1 μg of purified Spf1p in mixed detergent-lipid micelles, and 20 μg of total *S*. *cerevisiae* membranes containing Spf1p were treated with chymotrypsin for the time indicated on top of each lane. The reaction was stopped by the addition of 4 μg of aprotinin. The samples were run in a 9% SDS-PAGE and the Spf1p peptides detected by Western blot using an anti-Spf1p polyclonal antibody. *Lower panel*, 20 μg of total *S*. *cerevisiae* membranes containing GFP-Spf1p were treated with 0.5 μg of chymotrypsin for the time indicated on top of each lane. The samples were run in a 9% SDS-PAGE and the gel was scanned for GFP fluorescence.

Our preparation of purified Spf1p was obtained by the use of detergents which may lead to some protein denaturation and the creation of non-native susceptibility to proteolytic attack. With the aim of testing if this was the case of the observed Chy1 site in the detergent purified Spf1p, we investigated the effect of chymotrypsin digestion of Spf1p from isolated yeast membranes, and detected the proteolytic products by western blot using an anti-Spf1p antibody. As shown in Fig 4, while at zero time of proteolysis the antibody detected a major band corresponding to the full-length protein, after a short exposure to chymotrypsin the most intense band corresponded to the peptide generated by the Chy1 cut. The treatment with chymotrypsin of yeast membranes containing Spf1p generated a major proteolytic fragment similar to that produced from the purified Spf1p.

The proteolysis of the Spf1p in yeast membranes was also examined by following the fluorescent peptides produced by chymotrypsin digestion of the membrane inserted GFP-Spf1p. Despite the low total fluorescence intensity due to the low amounts of GFP-Spf1p present in the membranes loaded, two major proteolytic fluorescent products were detected, a larger one of Mr about 140 kDa and one of fast migration in the lower portion of gel. This proteolytic pattern is consistent with the cleavage of GFP-Spf1p at the Chy1 site and suggest that this site is similarly exposed in the membrane inserted Spf1p and in the purified Spf1p.

### Identification of the Spf1p chymotryptic cleavage sites by Edman sequencing and mass spectrometry

**Scheme 1:**
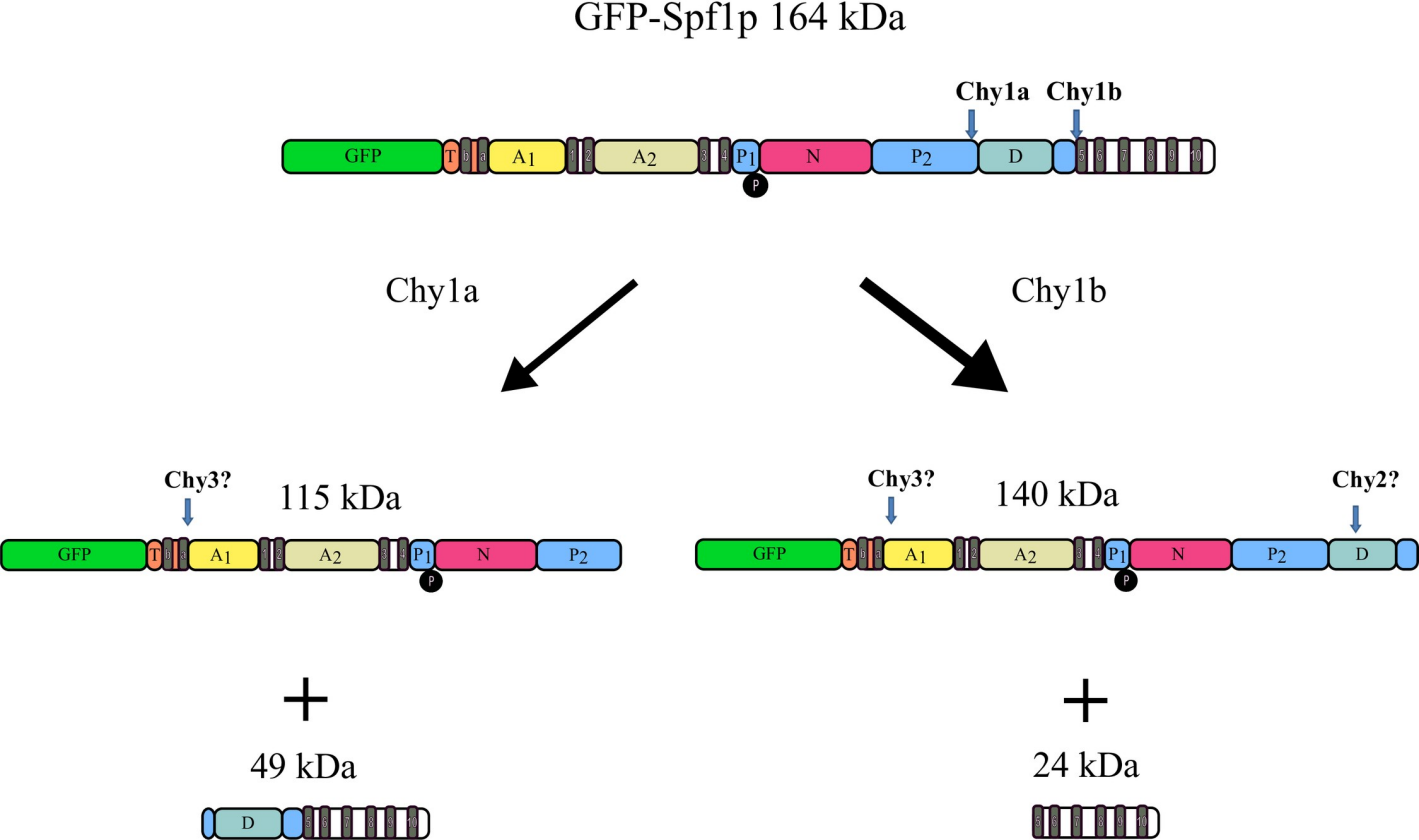
Schematic representation of the initial chymotryptic cleavage of the N-terminally-GFP fused Spf1p. Each cylinder represents a domain or subdomain of the protein. The length of the cylinder was made approximately proportional to its number of amino acid residues. *A*, *P*, *N*, P-ATPase actuator, phosphorylation and nucleotide domains. *T*, and *D*, P5A-ATPase unique P5A-ATPase regions. Transmembrane segments are represented as grey vertical cylinders, and the location of the catalytic phosphorylation is shown as a black sphere. The arrows indicate the initial chymotryptic cleavage sites *Chy1a* and *Chy1b*, and the possible locations of further proteolysis (*Chy2* and *Chy3*). The Mr indicated are those estimated from the amino acid sequence. The thicker arrow on the right is in accordance with the results showing that the most intense band produced by chymotryptic digestion producing the Chy1 fragment migrated as a peptide of about 140 kDa.

With the aim of identifying the sequence of the primary chymotryptic cleavage, the full-length GFP-Spf1p and the large chymotryptic fragment of 140 kDa were submitted to further in gel digestion with trypsin and the proteolytic peptides were analyzed by mass spectrometry. In the full-length GFP-Spf1p, 29 peptides were identified covering the whole protein from the N-terminal His-tag up to Spf1p residue Lys1211, just four residues upstream the C-terminus of the protein (S2 Fig). In agreement with the predicted topology of Spf1p, none of the identified peptides corresponded to putative Spf1p transmembrane segments. This is consistent with the hydrophobic nature of transmembrane residues which makes difficult to detect these fragments by conventional mass spectrometry. As expected from the in-gel fluorescence measurements, mass spectrometry analysis of the 140 kDa chymotryptic fragment showed the presence of peptides corresponding to the GFP, confirming that it originated from the cleavage of GFP-Spf1p at the C-terminal portion of the molecule. Moreover, one of the identified peptides had Arg979 at the C-terminus suggesting that the C-terminal end of the 140 kDa fragment was near transmembrane segment M5. We next digested GFP-Spf1p with chymotrypsin and submitted to amino terminal Edman sequencing the short proteolytic fragments that migrated between the Mr markers of 25 and 37 kDa. The sequence obtained was Ala-Leu-Asn-(X)-Leu, where (X) represents the amino acid from the fourth sequencing cycle which could not be identified. This amino acid sequence occurs twice in the structure of Spf1p, once in the C-terminal portion of the P-domain at position 769 where (X) is Ala772, and again in the cytosolic face of transmembrane segment M5 at position 996 where (X) is Cys999. We called these two putative chymotryptic cleavage sites Chy1a and Chy1b, respectively, but because of the uncertainty in the sequence obtained, it is possible that only one cut did actually occur. However, if there was only one cut at Chy1a site, the penultimate Ala should have been detected. Instead, we find more likely that the protein was cut both Chy1a and Chy1b sites, and the two observed sequences correspond to the N-terminal of different proteolytic fragments, close in size, that were not separated by the SDS-PAGE. If this was the case only in the fourth cycle, where the sequences at both sites differ from each other, the amino acid signal may have dropped below the detection limit. The fact that the main chymotryptic fragment produced had 140 kDa, suggests that the preferential chymotryptic pathway involved the primary cleavage at site Chy1b. Taking into account the estimated size of the fragments, the fluorescence and the mass spectrometry results, a possible pathway for the initial chymotryptic digestion of GFP-Spf1p is shown in Scheme 1.

### ATP binding protects Spf1p from chymotryptic cleavage at the Chy1

**Fig 5:**
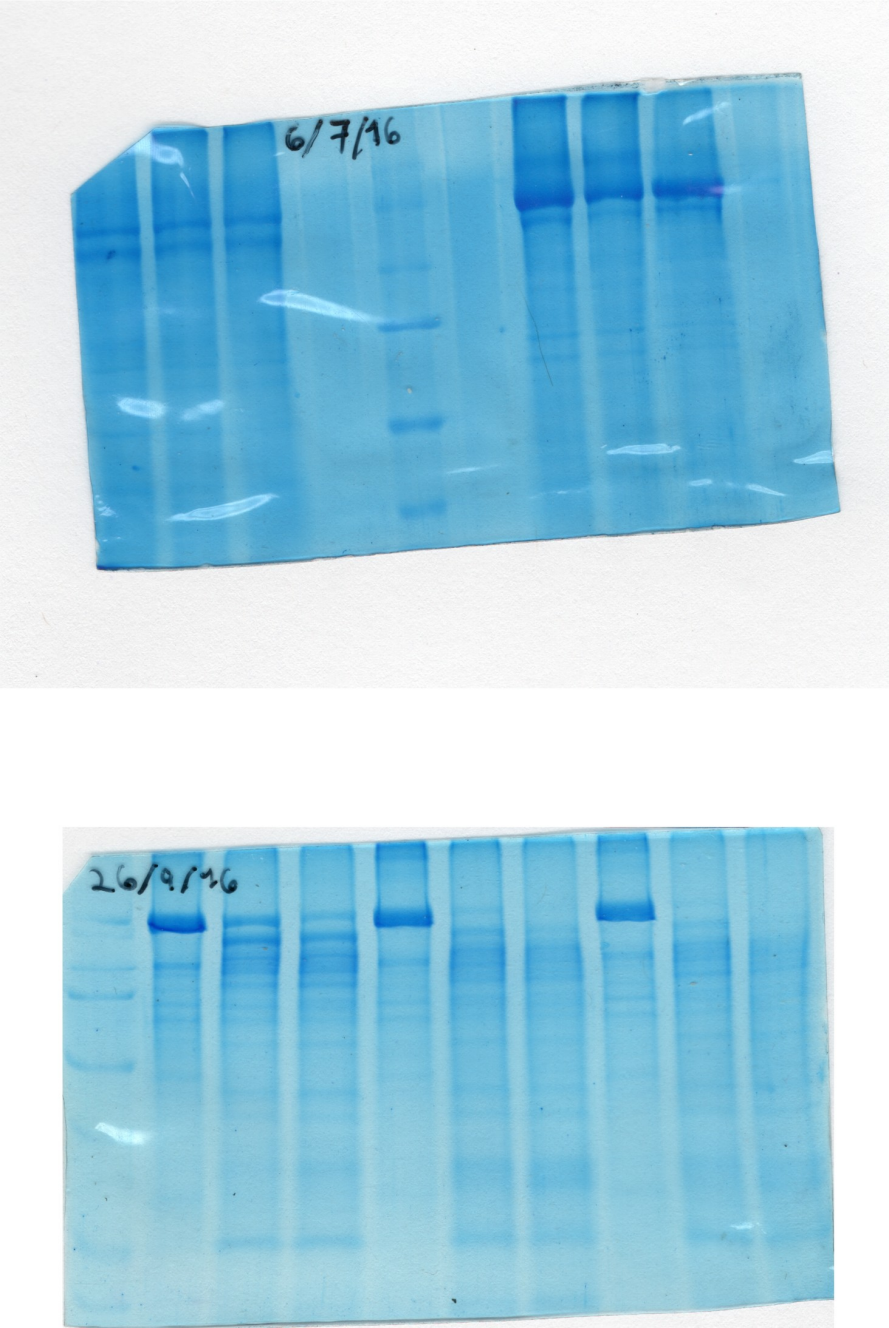
Effect of ATP on chymotryptic digestion of GFP-Spf1p. 3 μg of GFP-Spf1p was exposed to chymotrypsin (ratio w/w, 30:1) and the reaction was stopped by the addition of TCA. The composition of the proteolytic medium was *EDTA*, 2 mM EDTA, *Mg*, MgCl_2_ enough to give 2 mM free Mg^2+^, and *Ca*, CaCl_2_ enough to give 100 μM free Ca^2+^, each condition with or without 3 mM ATP. *Top panel*, the protein was digested for 30 min. *Bottom panel*, the proteolysis media were the same as described above and the numbers proteolysis time is indicated on top of each lane.

Previous studies of P-ATPases by limited proteolysis studies have documented discrete conformational states which could be stabilized by the binding of substrates and other ligands [30, 36–38]. In Fig 5 we examined the effect of Mg^2+^, Ca^2+^ and ATP on the chymotryptic digestion of Spf1p. In the presence of enough EDTA to remove free divalent cations from the proteolysis medium, the GFP-Spf1p was rapidly converted into the 140 kDa fragment by the cleavage at the Chy1 site. The presence of Mg^2+^ or Ca^2+^ in the proteolysis medium did not seem to change the proteolysis at the Chy1 site, and about half of the protein was digested to the 140 kDa product after 30 min. However, if the proteolysis medium contained ATP most of the protein was not digested indicating that the cleavage proceeded at a lower rate. Although some bands appeared to have different intensity, ATP did not change the observed general proteolytic pattern.

### Effect of proteolysis on the Spf1p functional state

**Fig 6:**
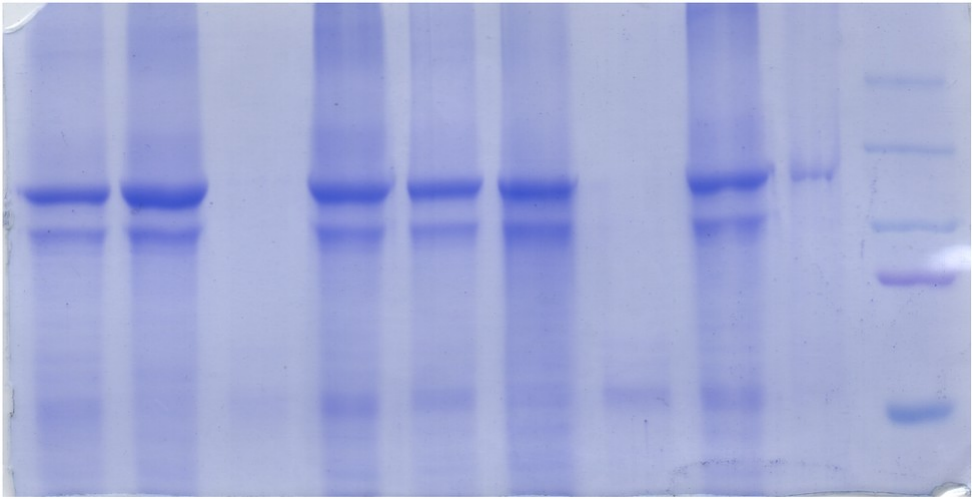
A. Effect of limited proteolysis on the ATPase activity of Spf1p. 4 μg of Spf1p from detergent-lipid micelles was digested for 30 min at 28°C with different amounts of the protease indicated in the figure. The chymotrypsin and trypsin digestions were stopped by the addition of 20 μg aprotinin, and proteinase K and V8 digestions were stopped by the addition of 0.5 mM PMSF. The ATPase activity was measured as described under “Materials and methods”. B. 10 μg of Spf1p in detergent micelles was exposed to chymotrypsin for different times, either in the absence *(-L)* or in the presence *(+L)* of PC. The proteolysis was ended by the addition of aprotinin and after the addition of PC to *(-L)* samples, the ATPase activity was measured. C. The Chy1 fragment does not dissociates from the micelle after proteolysis. 20 μg of Spf1p in detergent micelles was exposed to chymotrypsin for 5 min, either in the absence *(-L)* or in the presence *(+L)* of PC. After stopping the reaction an aliquot of the proteolyzed sample was saved (P), and the rest was incubated with 50 μl of Ni-NTA agarose, washed three times with 1 ml of solution containing 0, 25 and 50 mM imidazole and finally eluted with 1 ml of 500 mM imidazole. The protein from 100 μl of each step was precipitated with TCA, separated by SDS-PAGE and stained with Coomasie blue. *P*, proteolyzed sample before incubation with Ni-NTA resin, *FT*, supernatant containing part of the sample that did not bound to the Ni-NTA resin after the incubation, *W*, final wash with 50 mM imidazole, *E*, eluate of 500 mM imidazole. *C*, control of undigested Spf1p (0.75 μg), *M*, molecular weight markers.

The effect of limited proteolysis on the function of Spf1p was evaluated by measuring the residual ATPase activity. As shown in Fig 6, proteolysis of GFP-Spf1p with increasing concentrations of trypsin, proteinase K or V8 protease led to a progressive loss of function. Surprisingly, digestion with chymotrypsin minimally decreased the ATPase activity of GFP-Spf1p. The diverse response of Spf1p to proteolysis could be related to the fact that the proteases cleaved different regions of the Spf1p molecule, thus the cut by trypsin or proteinase K at the N-terminal region eliminated the ATPase activity, while the fragments produced by the cleavage of GFP-Spf1p by chymotrypsin were able to form a functional complex. We hypothesized that the 140 kDa and the C-terminal 25 kDa fragment produced by the GFP-Spf1p cut at Chy1 remained associated in the lipid-detergent micelles due to the hydrophobic nature of the transmembrane segment.

With the aim of decreasing the strength of the interaction the exposure of GFP-Spf1p to chymotrypsin was performed in the absence of lipids. The examination by SDS-PAGE of the proteolytic pattern indicated that similar peptides were produced either in the presence or in the absence of lipids. However, if lipids were absent during the proteolysis digestion the ATPase activity was rapidly lost.

**Fig 7:**
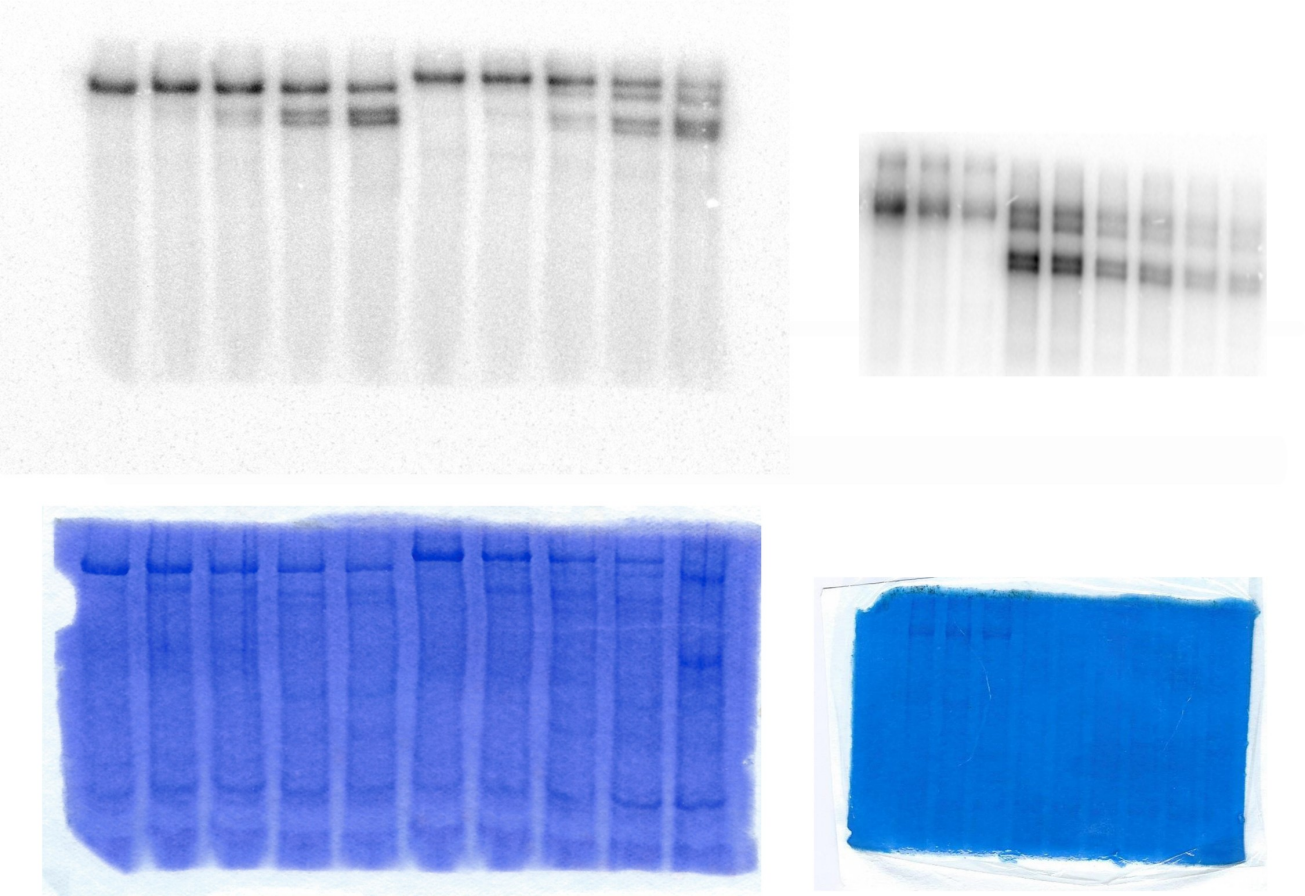
Formation and decomposition of the catalytic phosphoenzyme by the chymotrypsin treated Spf1p and Spf1p-GFP. The Spf1p and Spf1p-GFP proteins were digested with chymotrypsin at a (w/w) ratio of 30:1 protein to chymotrypsin. After the time indicated on top of each lane the proteolysis was stopped by adding 2.5 μg of aprotinin and cooling to 4°C. The phosphorylation reaction *(left panel)* was started by the addition of *0*.*5 μM [γ*^*32*^*P]-ATP* and terminated after 30 s by the addition of TCA. For dephosphorylation (*Dephos*, *right panel)* digested samples were phosphorylated for 60 s with *0*.*5 μM [γ*^*32*^*P]-ATP* and the dephosphorylation reaction started by the addition of 0.5 mM ATP. After the times indicated on top the reaction was stopped by the addition of TCA. The samples were electrophoresed in an acidic 7.5% SDS-PAGE and revealed by Colloidal Coomassie staining (*bottom panels)* and radioactivity (*top panels*). The migration of the Spf1p-GFP proteolytic product probably generated by the cleavage at the C-terminus of Spf1p is indicated with a star symbol (☆).

In order to investigate the interaction of the fragments after proteolysis we submitted a C-terminally His- tagged Spf1p to digestion with chymotrypsin and detected by SDS-PAGE the products that were able to bind to Ni-NTA resin. As shown in Fig 6C, despite the cleavage, the Chy1 fragment was present in the eluate indicating that it was still interacting with the short C-terminal peptide containing the His-tag. The presence or absence of lipids during the proteolysis reaction did not affect the recovery of the Chy1 fragment. The function of the Spf1p after proteolysis was analyzed further by looking at the formation and turnover of the catalytic phosphoenzyme. With this aim, Spf1p and Spf1p-GFP were digested with chymotrypsin and the proteolytic fragments were incubated with radiolabeled ATP. As shown in Fig 7, as digestion progressed three intense bands of phosphorylated peptides were detected, corresponding to the intact protein, and proteolytic fragments generated by the cut at Chy1b and Chy1a, in both Spf1p-GFP and Spf1p. In addition, proteolysis of Spf1p-GFP revealed an additional phosphorylated proteolytic fragment that exhibited a migration similar to that of the intact Spf1p. The analysis of this fragment by mass spectrometry indicated that it lacked GFP hence it may have originated by a chymotryptic cut near the original Spf1p C-terminus. As expected the phosphoproteins decayed rapidly after stopping the labeling with cold ATP and were sensitive to hydroxylamine confirming that they correspond to the catalytic aspartyl-phosphate phosphoenzyme (S3 Fig). Moreover, if lipids were not added during the proteolysis the ability of the proteolytic fragments to react with ATP to form the phosphoenzyme was greatly reduced (S4 Fig). Altogether, these results indicate that in lipid-detergent micelles the fragments generated by the cleavage of Spf1p at Chy1 site were still able to form a phosphorylated intermediate and hydrolyze ATP.

## Discussion

### The structure of Spf1p detected by limited proteolysis

Limited proteolysis is a well characterized strategy to gain information on the structure of proteins at a relatively low resolution and has been extensively used to advance the knowledge of different P-ATPases [29–42]. While the cleavage sites of each protease have specific primary sequence constrains, the precise location where the cuts occur are mostly determined by the segmental exposure and flexibility [27, 28]. We found that the exposure of Spf1p to proteases initially produced large fragments by trimming the N- and C- terminal regions. While trypsin and proteinase K apparently cleaved Spf1p at both ends, chymotrypsin exhibited an apparent preference for cleaving the C-terminal portion of Spf1p at a site that we called Chy1. Consistently with this idea, if the GFP was fused at the N-terminus of Spf1p a fluorescent chymotryptic product of 140 kDa was detected, while when the GFP was fused at the C-terminal end of Spf1p the main proteolytic products were not fluorescent and the fluorescence was observed in peptides that had a size near that of GFP. The migration in SDS-PAGE, the location of the GFP, and the mass spectrometry analysis of the proteolytic peptides were consistent with the location of Chy1 site in the P domain near the transmembrane segment M5. Further analysis by Edman sequencing indicated Tyr768-Ala769 and Leu995-Ala996 as possible locations for the Chy1 site. Tyr768 is in the C-terminal part of the P-domain and precedes the unique Spf1p segment that we called D region, while Leu995 is at the cytosolic end of M5, near residues that in other P-ATPases have been shown to bind the transported substrate [48].

Because the sites of limited proteolysis are characterized by their high chain flexibility, unstructured segments of the proteins are particularly susceptible to cleavage, and on the other hand, the presence of a cleavage site may indicate a region of a local unfolding. Previous limited proteolysis studies of P-ATPases have shown that some cleavages only occurs when high concentration of detergents and proteases are used which could lead to the exposure of regions of the protein that are not accessible in the native well folded state [49]. In this line, we found that chymotrypsin cut Spf1p at the Chy1 site both in the detergent-purified and in the membrane inserted forms of the protein, making unlikely that the observed proteolysis of Spf1p at Chy1 was the consequence of protein exposure to the detergents used for purification.

### The effect of ATP

We found that ATP added to the media of proteolysis slowed the cleavage of Spf1p by chymotrypsin. This effect did not require Mg^2+^ suggesting that it was independent of the turnover of the enzyme and was directly caused by the binding of the nucleotide to the Spf1p protein. The effect of ATP was rather surprising since the Chy1 chymotryptic site is not in the N domain, and did not involve a significant change in the size of the proteolytic products. We did not observe differences in the digestion pattern produced by ATP suggesting that no new cleavage sites were exposed. A similar effect of ATP slowing down the trypsinolysis of SERCA without changing the digestion pattern has been previously described and was attributed to the effect of the catalytic substrate on internal protein dynamics, rather than to a direct effect of the nucleotide on the site of proteolytic cleavage [38]. Following this interpretation, our results would suggests that the binding of ATP to the N domain stabilized a more ordered structure of Spf1p, limiting the accessibility of the protease to the Chy1 on the downstream C-terminal part of the P domain.

### Functional effects of limited proteolysis on the Spf1p

Proteolysis of GFP-Spf1p with trypsin or proteinase K rapidly inactivated the enzyme concomitantly with the production of major non-fluorescent fragments of about 100 kDa. This pattern suggests that trypsin or proteinase K cleaved the C-terminal region plus the GFP and a N-terminal segment of Spf1p that was critical for the function or stability of Spf1p. In contrast, we found that the mixture of chymotryptic fragments produced by Spf1p was still capable of ATP hydrolysis and catalytic phosphoenzyme turnover. This finding suggests that the fragments of about 140 kDa and 25 kDa generated by the split of GFP-Spf1p at Chy1 were able to interact to produce a functional enzyme. Consistently with this idea we found that the Chy1 fragment was retained by the Ni-NTA matrix despite not having a His-tag. This is not surprising since we have previously reported a similar functional interaction between fragments containing transmembrane segments after proteolysis of the plasma membrane Ca^+^ pump [50].

Similar to other P-ATPases, lipids are necessary for the function or stability of Spf1p [24]. However, we found that the presence of lipids was specifically required during the chymotrypsin proteolysis for the preservation of the ATPase activity, and they were no longer effective if added to the mixture after proteolysis. The analysis by SDS-PAGE did not reveal substantial differences between the proteolytic fragments, either with or without lipids. This result indicates that lipids have a subtle effect on the structure and function of Spf1p and will require further investigation.

### Comparison of the limited proteolysis pattern of Spf1p with that of other P-ATPases

The structure of several P-ATPases have been thoroughly examined by limited proteolysis, in particular that of the Na^+^/K^+^ ATPase, SERCA and those like PMCA or Drs2p which have flexible terminal regulatory domains [29–42]. These studies show that despite the differences in primary sequence, the preferential proteolytic attack occur in well-defined regions, as it would be expected from sharing a similar structure and domain organization [48, 49]. The primary cleavages are generally located at the N-terminal segment preceding M1, the cytosolic loops between M2 and M3, the large soluble segment encompassing transmembrane segments M4-M5 and near the C-terminus. These sites are differentially exposed in specific P-ATPase conformations, and in particular those in the M2-M3 loop are highly exposed in the E_2_ conformation [30, 35–37]. Unfortunately, our results did not show definitive evidence of the proteolytic cleavage of Spf1p in the M2-M3 region of the protein, which may indicate that in our proteolysis medium the M2-M3 loop of Spf1p is not preferentially exposed. It is worth to note that, in the experimental conditions we used for proteolysis the Spf1p protein is capable of reacting with ATP to form the catalytic phosphoenzyme which suggest that rapidly adopts the E_1_ conformation even in the absence of the transported substrate.

Various protease susceptible sites have been detected in the large M4-M5 loop of different P-ATPases. The Na^+^/K^+^ ATPase stabilized in the E_2_ conformation is cleaved by trypsin preferentially at Arg438 (T1 site) and subsequently at Arg589 (T4 site) near the boundaries of the insertion of the N domain into the P domain [29, 30]. Similarly, SERCA is cut by trypsin at Arg505, just before the characteristic N-domain motif KGAPE, while other proteases like proteinase K, exhibit two cleavage sites at Ser350 near the phosphate-acceptor aspartate, and at the C-terminal boundary of the N-domain [35]. In contrast, our results suggest that Spf1p is cleaved by chymotrypsin primarily in the M4-M5 loop C-terminal portion corresponding to the P-domain near M5. This is somehow unexpected since the cleavage of other P-ATPases in this region only takes place after extensive proteolytic digestion [34, 51]. Indeed, the trypsin cut of the Na^+^/K^+^ ATPase at Gln737 near the amino terminal end of the cytosolic portion of M5 helix occurs only after a long exposure to the protease and generates the so-called protease-resistant K^+^-occluding 19 kDa membranes [34]. Noteworthy, the stabilization of the 19 kDa Na^+^/K^+^ ATPase membranes requires the presence of the transported ion (K^+^) while in its absence a proteolytic fragment containing M5-M6 hairpin is spontaneously released from the membrane [51]. Taking in account this data, an interesting possibility is that the propensity to chymotryptic cleavage of Spf1p near M5 that we observed may be affected by the binding of the transported substrate.

### Characteristics of the Spf1p P5A-ATPase near transmembrane segment M5

The amino acid sequence of Spf1p shows high similarity with that of other P-ATPases in specific regions of well-known function like the actuator (A), the nucleotide-binding (N) and the phosphorylation (P) domains. A unique feature of P5A-ATPases is the presence of an extended linker that connects the C-terminus of the P domain with the long helix that becomes transmembrane segment M5, and that we called D region. Multiple sequence alignment analysis shows that this stretch of amino acids it is not well conserved among different species and its length is variable, from very short as in the P5A-ATPase from *Trichomonas vaginalis* with only 12 residues, or as long as 131 residues in the P5A-ATPase from *Symbiodinium microadriaticum* (dinoflagellate). The structural analysis of the amino acid sequence predicts that at least part of this segment is disordered, a fact that may explain its relative low sequence conservation and susceptibility to proteolytic attack. Although there is no information available about the importance of this sequence region D, it has been shown that despite the variability in the D region, P5A-ATPases from different species can rescue the yeast Spf1p K.O phenotype [2]. Although this fact indicates that the full D region is not essential for function, this segment may be involved the regulation of Spf1p function as suggested by its optimal location as a hinge between domain P and M5.

In Spf1p, the region D is followed by a relatively well conserved segment that corresponds to the helical cytosolic extension of M5, and has been previously defined as P-ATPase motif “h” [52]. The rotation and movement of the stalk region of M5 comprising motif “h” has been shown to be a key step in the coupling of ATP hydrolysis and substrate transport by the P-ATPases [37].

The Spf1p M5 exhibits a poor identity with the corresponding segment of other P-ATPases and it has specific characteristics that may be related to its susceptibility to proteolytic cleavage (S5 Fig). Although Spf1p residues Lys993 and Asn998 near the cytosolic face are highly conserved, the M5 of P5A-ATPases does not contain negatively charged residues that could coordinate a putative cation substrate as is the case of Asn768 and Glu771 of SERCA. Recently, the elucidation of the structures of P4-ATPases and studies of mutagenesis has revealed the importance of the M5 residue Lys850 (in ATP8A1) in the lipid flipping-coupled dephosphorylation reaction [48, 53]. Interestingly, Lys850 from ATP8A1, and the transport ion coordinating residue Ser775 from the Na^+^ K^+^-ATPase, align with Spf1p Asn998, which is located just a few residues downstream the chymotryptic cleavage site we identified as Chy1b. Another characteristic of M5 Spf1p that can be noted is that even though its total hydrophobicity is similar to that from other P-ATPases, it is less amphipathic. Indeed, in the M5 of SERCA, Na^+^/K^+^ ATPase and other P-ATPases, a hydrophobic face opposed to the substrate face can be easily identified, while in contrast the homology modelling of Spf1p M5 suggests that the candidate substrate interacting residue Asn998 is surrounded by hydrophobic residues. While this observation may arise from the lack of a good template for homology modelling, alternatively it could indicate that the binding of the substrate to M5 Spf1p involve polar and hydrophobic interactions.

The M5, together with M2, M4 and M6 are internal helices that constitute the core of the transport domain in the P-ATPases. In the P5-ATPases the M4 segment exhibits the unique motif PPxxPxx, that in Spf1p as in other P5A-ATPases is PP(E/D)LPxE. The presence of the first proline of this motif is conserved in all P-ATPases and it discontinuous the M4 helix to expose ion-coordinating residues. It is interesting to consider that the presence of the second proline in the M4 of P5-ATPases would further distort M4 helix and, in the case of the P5A subgroup will contribute to the displacement of the two negatively charged glutamate residues toward the cytosolic face of M4. Altogether, the properties of Spf1p M4 and M5 segments and the flexibility generated by the D region are suggestive of a permissive cavity in that may accommodate large substrate. These particular properties of Spf1p and the vacancy of the putative substrate at the transport site may allow the chymotrypsin to get near the membrane to a site that would expected, a priory, inaccessible to proteases. The peptidyl nature of the substrate of P5A-ATPases proposed recently during the revision of this manuscript by McKenna et al., 2020, is in well agreement with these predictions.

## Supporting information

S1 Fig 1.5 μg of GFP-Spf1p was treated with chymotrypsin at GFP-Spf1p: protease ratio (*w/w*) 20:1 as indicated in “Materials and methods”. After the time indicated on top of each lane the proteolysis was stopped by the addition of 3 μg of aprotinin. The samples were submitted to SDS-PAGE on a 10% gel and stained with Colloidal Coomassie Blue and GFP fluorescence measurement. Notice that in order to preserve GFP fluorescence samples are not fully denatured and thus the migration of the peptides may not fully agree with the Mr.
(TIF)

S2 Fig Mass spectrometry analysis and Edman degradation sequencing. The amino acid sequence of the His-tagged GFP-Spf1p is shown. The sequences of peptides identified by mass spectrometry after “in gel” trypsinolysis are indicated in italics (*green*, GFP, *yellow*, Spf1p). The predicted transmembrane segments are indicated with bold letters. *On top*, analysis of the full-length GFP-Spf1p, and *on the bottom*, that of the 140 kDa fragment obtained by chymotrypsin treatment of GFP-Spf1p. The sequences that agree with the results of the Edman degradation of chymotrypsin digested GFP-Spf1p are boxed (*pink*).
(TIF)

S3 Fig Hydroxylamine sensitivity of the phosphorylated Spf1p-GFP chymotryptic fragments. Spf1p-GFP was digested for the time indicated on top of each lane and phosphorylated as described in Fig 7. The phosphoproteins were treated with 300 mM hydroxylamine for 10 minutes at 20°C, precipitated with TCA and separated in an acidic SDS-PAGE.
(TIF)

S4 Fig Formation of the catalytic phosphoenzyme by the Spf1p-GFP after proteolysis with chymotrypsin in the presence or absence of lipids. Spf1p-GFP in detergent micelles either with (*+L)* or without *(-L)* of PC was exposed to chymotrypsin (protein: protease ratio 20:1) for the time indicated on top of each lane. The proteolysis was ended by the addition of aprotinin and after the addition of PC, *[γ*^*32*^*P]-ATP* was added and the phosphorylation reaction was carried out for 30 s. The samples were electrophoresed in an acidic SDS-PAGE and the radioactive fragments were detected.
(TIF)

S5 Fig Panel A. Alignments of cytosolic and transmembrane helix M5. The amino acid sequences of yeast Spf1p, human ATP13A1, yeast Drs2p, human ATP8A1, pig Na^+^/K^+^-ATPase, and rabbit SERCA1 are shown. At the bottom, the helix representation of the transmembrane segments obtained by using the program HeliQuest [54]. The arrow in the center of the helix indicates the direction and magnitude of the hydrophobic moment of the helix. Panel B. Homology modeling of Spf1p M5 transmembrane segment. Representation of the surface of M5 of the indicated P-ATPase obtained using the Chimera software and colored according the Kyte-Doolitle hydrophobicity scale (*red*, hydrophobic, *blue*, polar). The structures are Drs2 *6PSX*, ATP8A1 *6K7G*, Na^+^/K^+^-ATPase *4HQJ* and Serca1 *4H1W*. Homology models for M5 of Spf1p and ATP13A1 were obtained using Phyre2 [55]. The structures were aligned using the matchmaker structure comparison command from Chimera and are presented showing the polar substrate-binding residues facing forward.
(TIF)

## Abbreviations

Spf1p: sensitivity to *Pichia farinosa* killer toxin protein
ER: endoplasmic reticulum
C_12_E_10_: polyoxyethylene 10-lauryl ether; SDS-PAGE, sodium dodecyl sulfate-polyacrylamide gel electrophoresis
PC: phosphatidylcholine
TCA: trichloroacetic acid
