# Correction: Highly exposed segment of the Spf1p P5A-ATPase near transmembrane M5 detected by limited proteolysis

**DOI:** 10.1371/journal.pone.0256945

**Published:** 2021-08-26

**Authors:** The PLOS ONE Staff

Incorrect versions of the figures were published. The authors have provided corrected versions here. The publisher apologizes for the errors.

**Fig 1:**
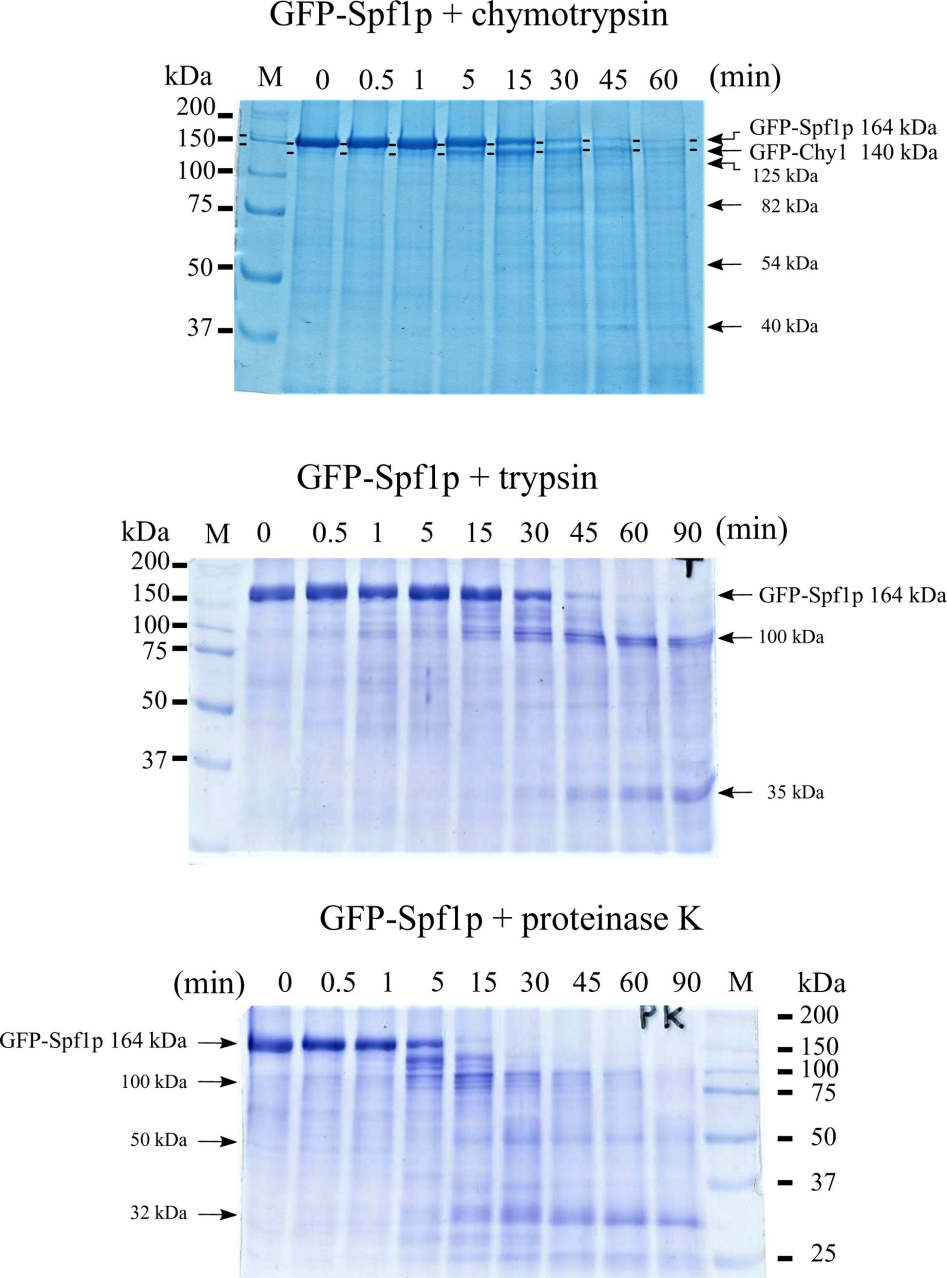
Limited proteolysis of GFP-Spf1p. 2.5 μg of GFP-Spf1p was treated with the indicated protease as described under “Materials and methods”. After the time indicated on top of each lane the proteolysis was stopped by the addition of TCA and the samples submitted to SDS-PAGE on a 9% gel and stained with Colloidal Coomassie Blue. GFP-Spf1p: protease ratio (*w/w*) was 20:1 (A) chymotrypsin, (B), trypsin, (C), proteinase K. The “0” min were processed adding TCA before the protease. The arrows indicate the estimated Mr according to the migration.

**Fig 2:**
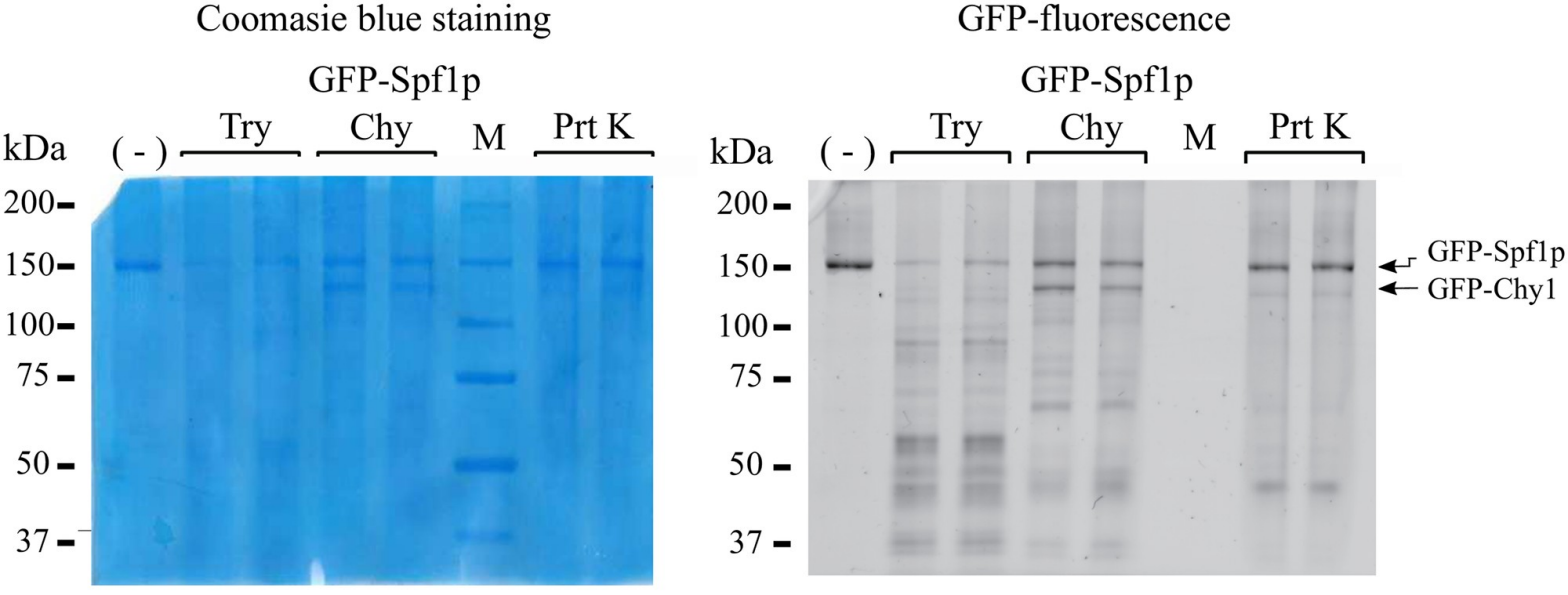
In-gel fluorescence analysis of the GFP-Spf1 digests. 1.5 μg GFP-Spf1 was exposed for 5 min at 28°C to the action of the indicated protease as described under “Materials and methods”. GFP-Spf1p: protease ratio *(w/w)* was *Try*, trypsin: (5:1), *Chy*, chymotrypsin (20:1) and *Prt K*, proteinase K (20:1), and the reaction was stopped by the addition of 12 μg or 3 μg of aprotinin for trypsin and chymotrypsin respectively. Proteinase K digestion was stopped by the addition of 0.5 mM PMSF. The panel on the left shows the Coomasie blue stained gel and on the right the measurement of GFP fluorescence. Two lanes from duplicate proteolysis reactions for each protease are shown.

**Fig 3:**
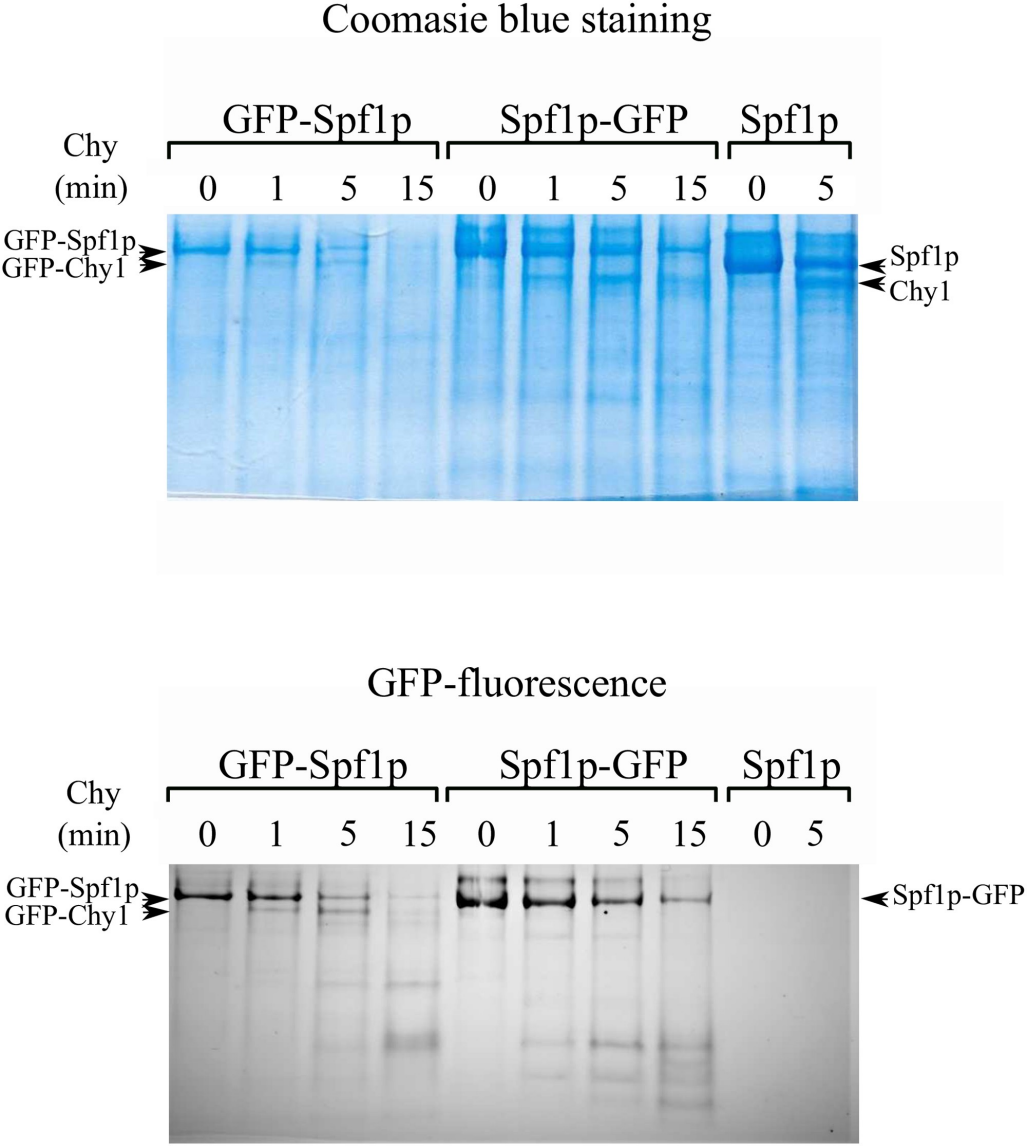
Comparison of the chymotryptic digestion pattern of GFP-Spf1, Spf1p-GFP and Spf1p. Spf1p and the Spf1p GFP fusion protein indicated in the figure were digested with chymotrypsin at a ratio protein: protease (w/w) of 20:1. After the time indicated on top of each lane, the proteolysis was stopped by adding 5 μg of aprotinin. The samples were electrophoresed in a 9% SDS-PAGE and revealed by Colloidal Coomassie staining (*left panel*) or GFP fluorescence (*right panel*). The arrows indicate the migrations of the GFP-Spf1p fragments produced by the cut at Chy1 and the equivalent non-fluorescent fragment generated from Spf1p-GFP and Spf1p.

**Fig 4:**
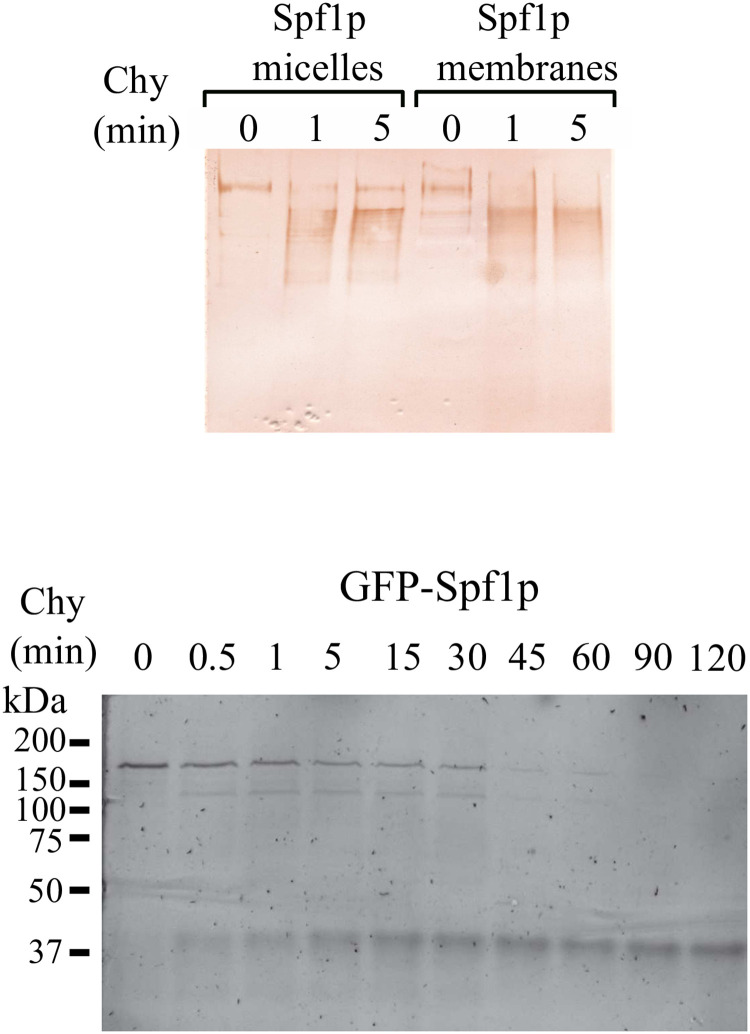
Chymotryptic digestion of detergent-purified Spf1p and Spf1p from yeast membranes. *Top panel*, 0.1 μg of purified Spf1p in mixed detergent-lipid micelles, and 20 μg of total *S*. *cerevisiae* membranes containing Spf1p were treated with chymotrypsin for the time indicated on top of each lane. The reaction was stopped by the addition of 4 μg of aprotinin. The samples were run in a 9% SDS-PAGE and the Spf1p peptides detected by Western blot using an anti-Spf1p polyclonal antibody. *Lower panel*, 20 μg of total *S*. *cerevisiae* membranes containing GFP-Spf1p were treated with 0.5 μg of chymotrypsin for the time indicated on top of each lane. The samples were run in a 9% SDS-PAGE and the gel was scanned for GFP fluorescence.

**Fig 5:**
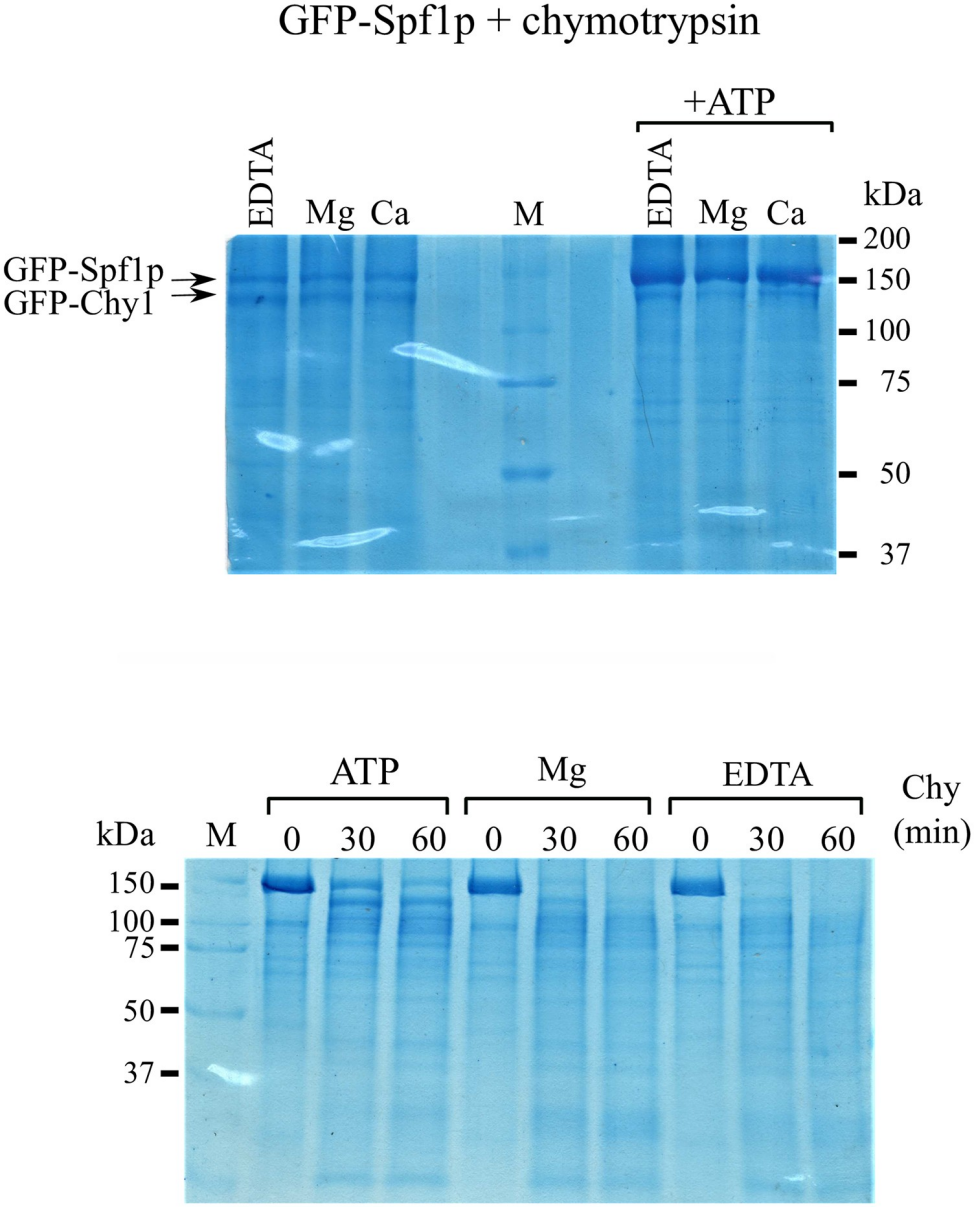
Effect of ATP on chymotryptic digestion of GFP-Spf1p. 3 μg of GFP-Spf1p was exposed to chymotrypsin (ratio w/w, 30:1) and the reaction was stopped by the addition of TCA. The composition of the proteolytic medium was *EDTA*, 2 mM EDTA, *Mg*, MgCl_2_ enough to give 2 mM free Mg^2+^, and *Ca*, CaCl_2_ enough to give 100 μM free Ca^2+^, each condition with or without 3 mM ATP. *Top panel*, the protein was digested for 30 min. *Bottom panel*, the proteolysis media were the same as described above and the numbers proteolysis time is indicated on top of each lane.

**Fig 6:**
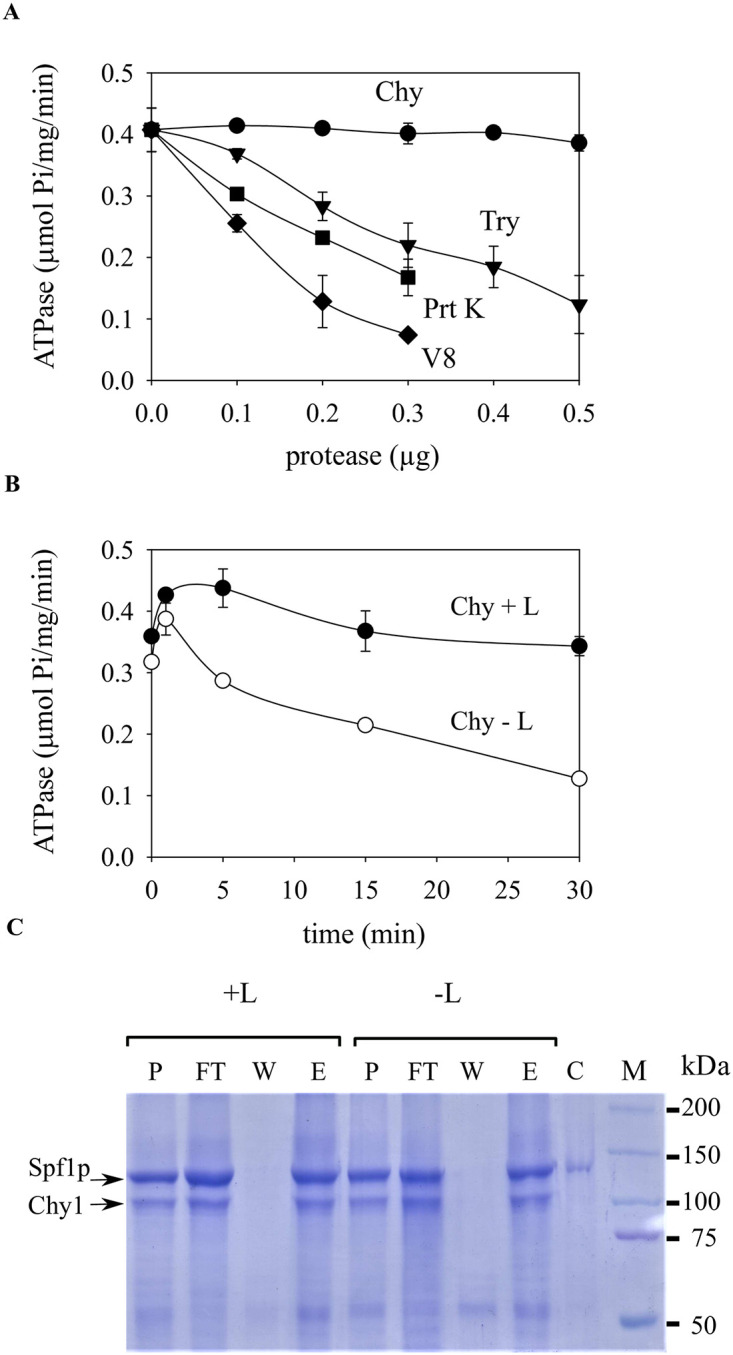
A. Effect of limited proteolysis on the ATPase activity of Spf1p. 4 μg of Spf1p from detergent-lipid micelles was digested for 30 min at 28°C with different amounts of the protease indicated in the figure. The chymotrypsin and trypsin digestions were stopped by the addition of 20 μg aprotinin, and proteinase K and V8 digestions were stopped by the addition of 0.5 mM PMSF. The ATPase activity was measured as described under “Materials and methods”. B. 10 μg of Spf1p in detergent micelles was exposed to chymotrypsin for different times, either in the absence *(-L*) or in the presence *(+L)* of PC. The proteolysis was ended by the addition of aprotinin and after the addition of PC to *(-L)* samples, the ATPase activity was measured. C. The Chy1 fragment does not dissociates from the micelle after proteolysis. 20 μg of Spf1p in detergent micelles was exposed to chymotrypsin for 5 min, either in the absence *(-L*) or in the presence *(+L)* of PC. After stopping the reaction an aliquot of the proteolyzed sample was saved (P), and the rest was incubated with 50 μl of Ni-NTA agarose, washed three times with 1 ml of solution containing 0, 25 and 50 mM imidazole and finally eluted with 1 ml of 500 mM imidazole. The protein from 100 μl of each step was precipitated with TCA, separated by SDS-PAGE and stained with Coomasie blue. *P*, proteolyzed sample before incubation with Ni-NTA resin, *FT*, supernatant containing part of the sample that did not bound to the Ni-NTA resin after the incubation, *W*, final wash with 50 mM imidazole, *E*, eluate of 500 mM imidazole. *C*, control of undigested Spf1p (0.75 μg), *M*, molecular weight markers.

**Fig 7:**
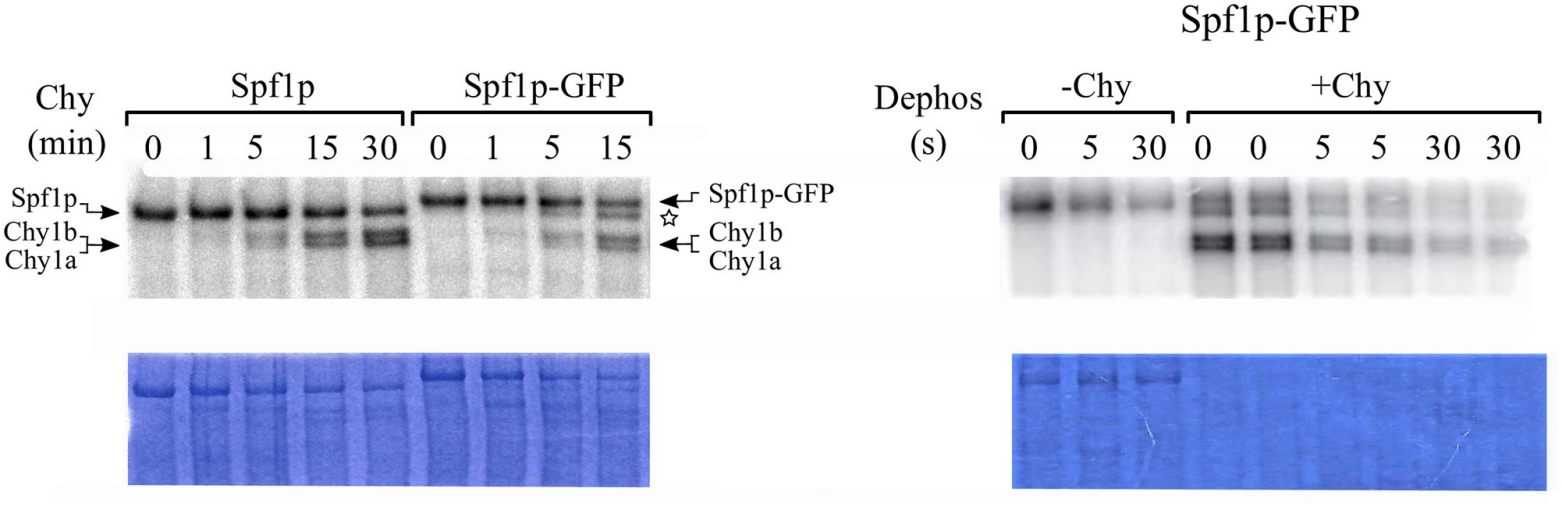
Formation and decomposition of the catalytic phosphoenzyme by the chymotrypsin treated Spf1p and Spf1p-GFP. The Spf1p and Spf1p-GFP proteins were digested with chymotrypsin at a (w/w) ratio of 30:1 protein to chymotrypsin. After the time indicated on top of each lane the proteolysis was stopped by adding 2.5 μg of aprotinin and cooling to 4°C. The phosphorylation reaction *(left panel*) was started by the addition of *0*.*5 μM [γ*^*32*^*P]-ATP* and terminated after 30 s by the addition of TCA. For dephosphorylation (*Dephos*, *right panel*) digested samples were phosphorylated for 60 s with *0*.*5 μM [γ*^*32*^*P]-ATP* and the dephosphorylation reaction started by the addition of 0.5 mM ATP. After the times indicated on top the reaction was stopped by the addition of TCA. The samples were electrophoresed in an acidic 7.5% SDS-PAGE and revealed by Colloidal Coomassie staining (*bottom panels*) and radioactivity (*top panels*). The migration of the Spf1p-GFP proteolytic product probably generated by the cleavage at the C-terminus of Spf1p is indicated with a star symbol (☆).
