# Supplementary figures and images for: Highly exposed segment of the Spf1p P5A-ATPase near transmembrane M5 detected by limited proteolysis
Source: PLoS One. 2021 Jan 28;16(1):e0245679. doi: 10.1371/journal.pone.0245679 (PMC7842927; doi:10.1371/journal.pone.0245679)

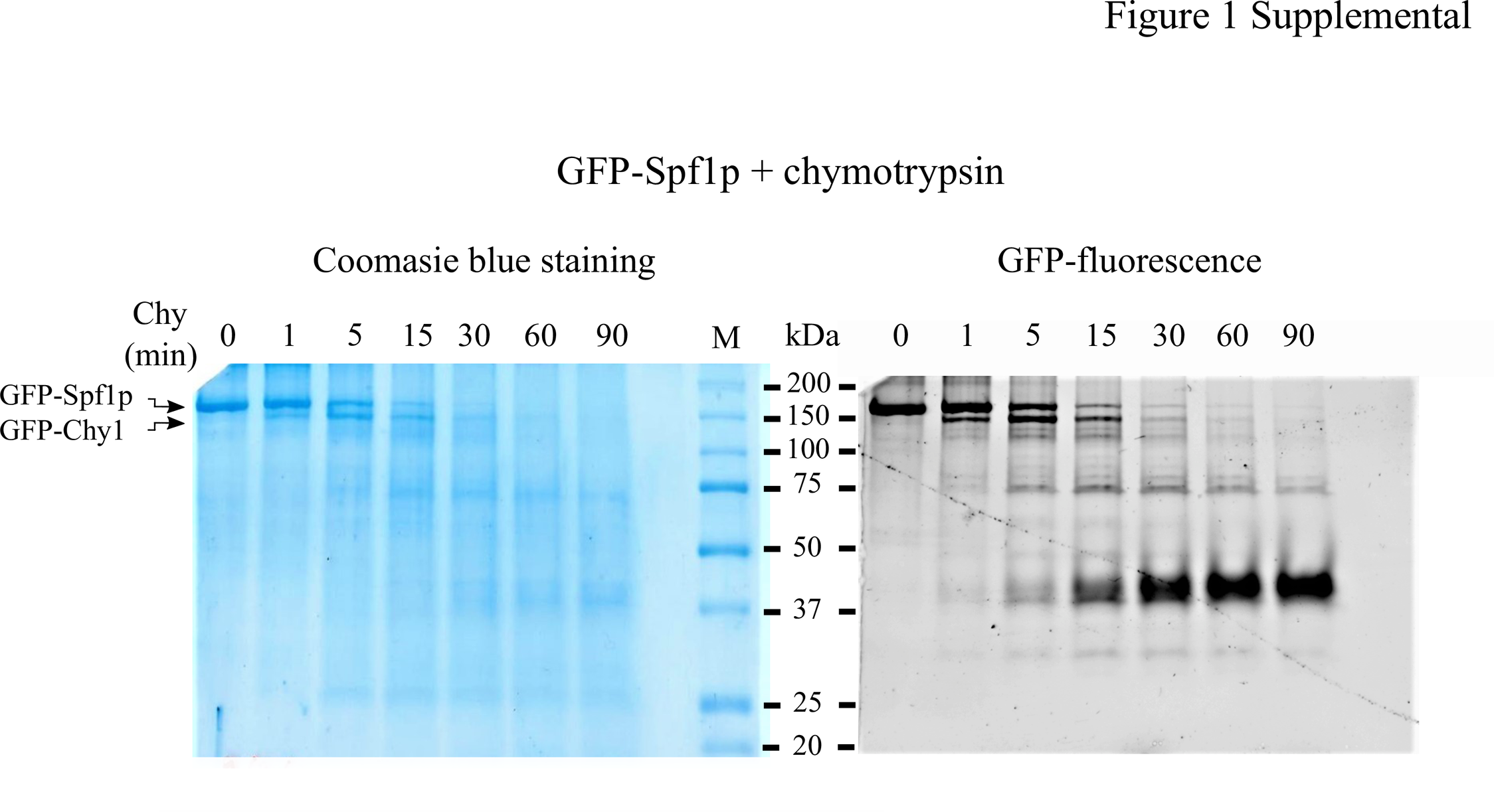

Supplement: S1 Fig — After the time indicated on top of each lane the proteolysis was stopped by the addition of 3 μg of aprotinin. The samples were submitted to SDS-PAGE on a 10% gel and stained with Colloidal Coomassie Blue and GFP fluorescence measurement. Notice that in order to preserve GFP fluorescence samples are not fully denatured and thus the migration of the peptides may not fully agree with the Mr. (TIF) [file pone.0245679.s001.tif]

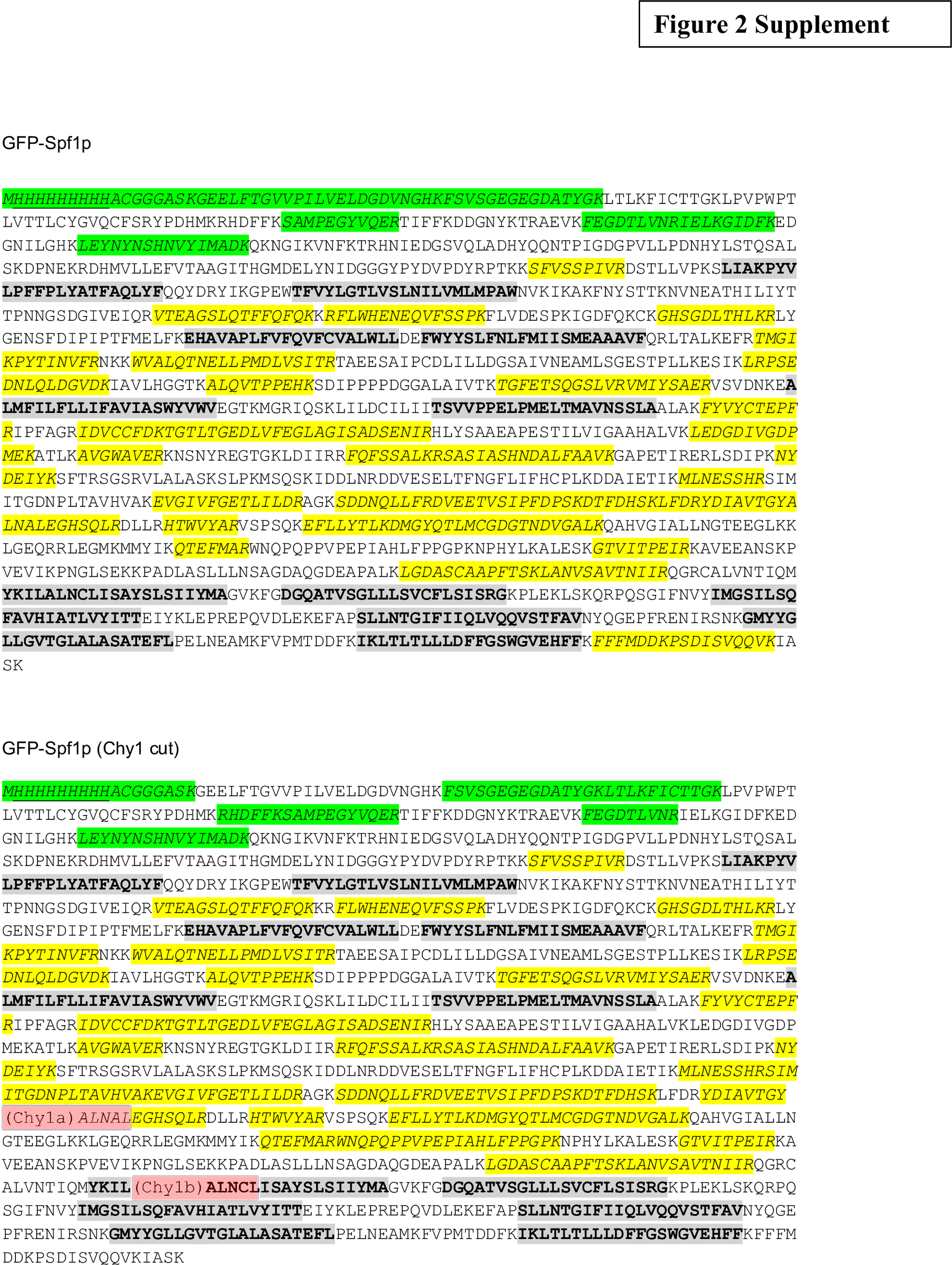

Supplement: S2 Fig — The amino acid sequence of the His-tagged GFP-Spf1p is shown. The sequences of peptides identified by mass spectrometry after “in gel” trypsinolysis are indicated in italics (green, GFP, yellow, Spf1p). The predicted transmembrane segments are indicated with bold letters. On top, analysis of the full-length GFP-Spf1p, and on the bottom, that of the 140 kDa fragment obtained by chymotrypsin treatment of GFP-Spf1p. The sequences that agree with the results of the Edman degradation of chymotrypsin digested GFP-Spf1p are boxed (pink). (TIF) [file pone.0245679.s002.tif]

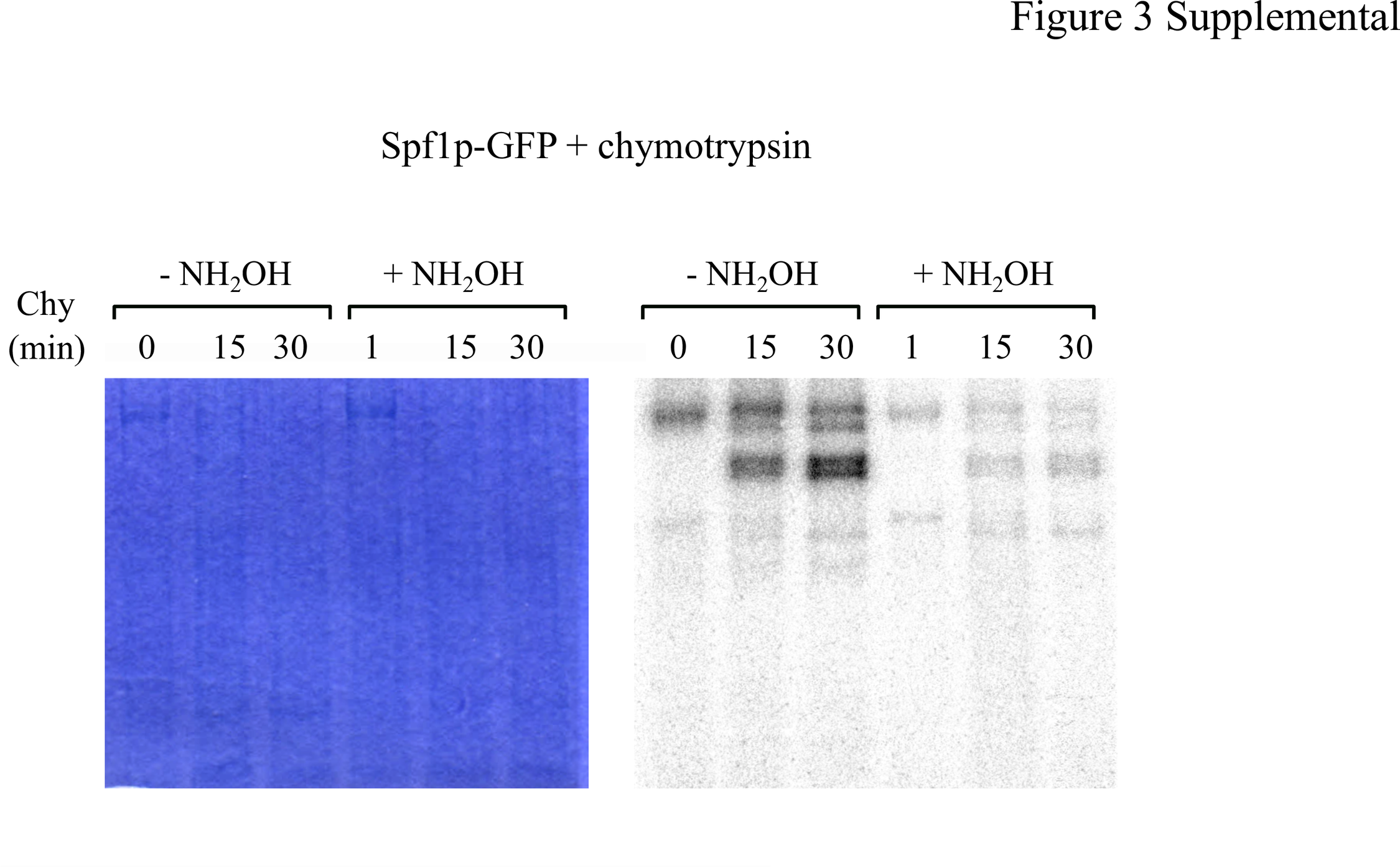

Supplement: S3 Fig — Spf1p-GFP was digested for the time indicated on top of each lane and phosphorylated as described in Fig 7. The phosphoproteins were treated with 300 mM hydroxylamine for 10 minutes at 20°C, precipitated with TCA and separated in an acidic SDS-PAGE. (TIF) [file pone.0245679.s003.tif]

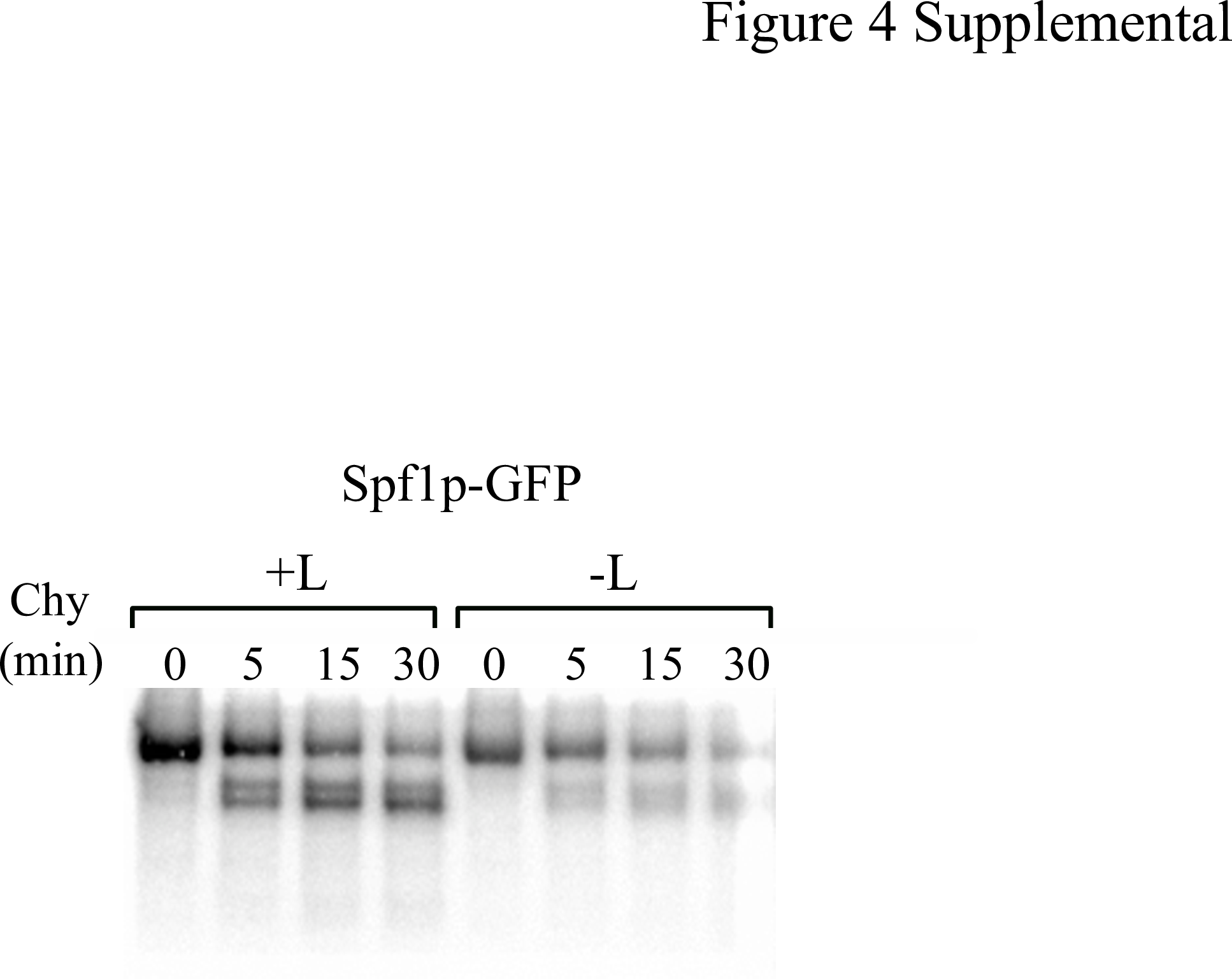

Supplement: S4 Fig — Spf1p-GFP in detergent micelles either with (+L) or without (-L) of PC was exposed to chymotrypsin (protein: protease ratio 20:1) for the time indicated on top of each lane. The proteolysis was ended by the addition of aprotinin and after the addition of PC, [γ32P]-ATP was added and the phosphorylation reaction was carried out for 30 s. The samples were electrophoresed in an acidic SDS-PAGE and the radioactive fragments were detected. (TIF) [file pone.0245679.s004.tif]

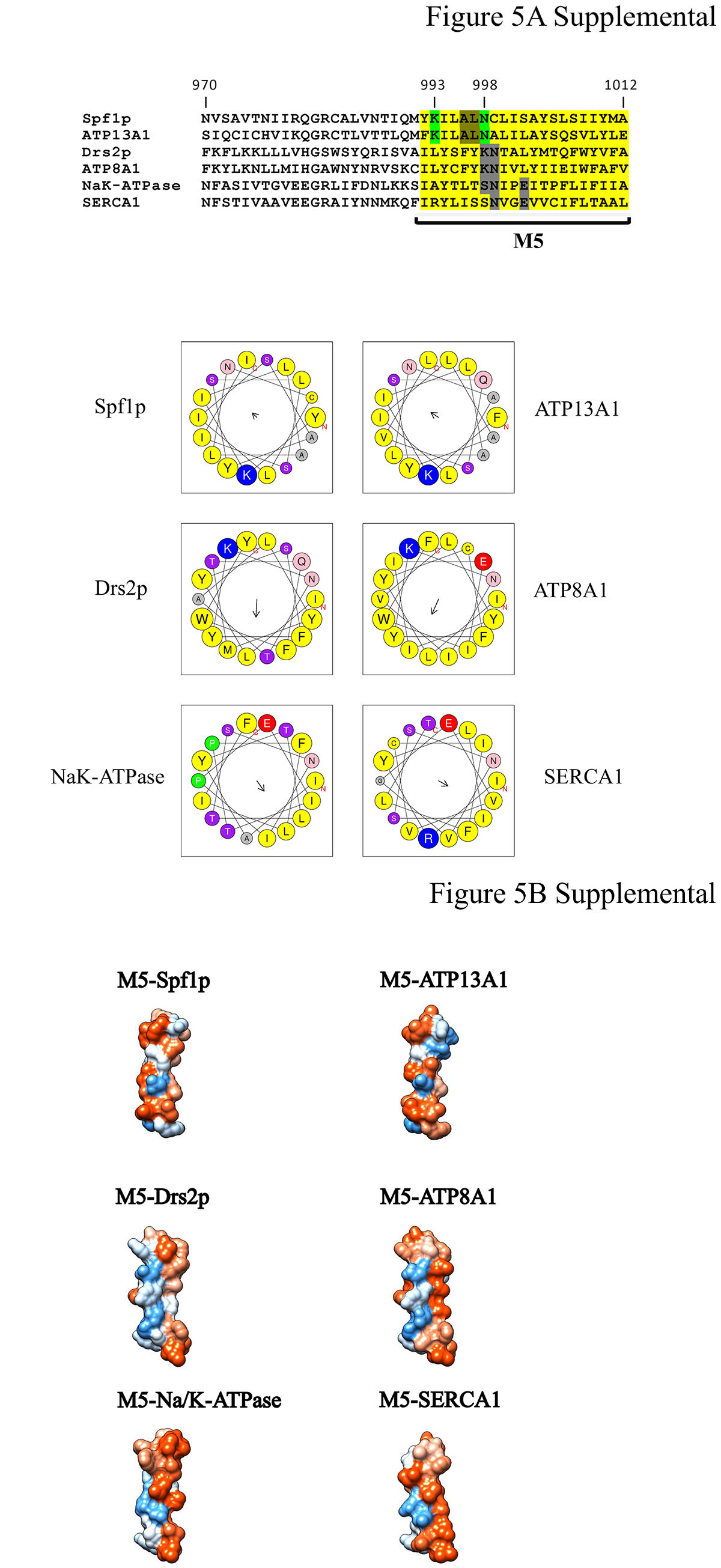

Supplement: S5 Fig — Panel A. Alignments of cytosolic and transmembrane helix M5. The amino acid sequences of yeast Spf1p, human ATP13A1, yeast Drs2p, human ATP8A1, pig Na+/K+-ATPase, and rabbit SERCA1 are shown. At the bottom, the helix representation of the transmembrane segments obtained by using the program HeliQuest [54]. The arrow in the center of the helix indicates the direction and magnitude of the hydrophobic moment of the helix. Panel B. Homology modeling of Spf1p M5 transmembrane segment. Representation of the surface of M5 of the indicated P-ATPase obtained using the Chimera software and colored according the Kyte-Doolitle hydrophobicity scale (red, hydrophobic, blue, polar). The structures are Drs2 6PSX, ATP8A1 6K7G, Na+/K+-ATPase 4HQJ and Serca1 4H1W. Homology models for M5 of Spf1p and ATP13A1 were obtained using Phyre2 [55]. The structures were aligned using the matchmaker structure comparison command from Chimera and are presented showing the polar substrate-binding residues facing forward. (TIF) [file pone.0245679.s005.tif]
